# Simulation studies, 3D QSAR and molecular docking on a point mutation of protein kinase B with flavonoids targeting ovarian Cancer

**DOI:** 10.1186/s40360-021-00512-y

**Published:** 2021-11-02

**Authors:** Suchitra Maheswari Ajjarapu, Apoorv Tiwari, Gohar Taj, Dev Bukhsh Singh, Sakshi Singh, Sundip Kumar

**Affiliations:** 1 Bioinformatics Sub-DIC, Department of Molecular Biology & Genetic Engineering, College of Basic Science and Humanities, Govind Ballabh Pant University of Agriculture and Technology, Pantnagar, Udham Singh Nagar, 263145 Uttarakhand India; 2 Department of Biotechnology, Andhra University, Vishakhapatnam, 530003 Andhra Pradesh India; 3 Department of Computational Biology and Bioinformatics, Jacob Institute of Biotechnology and Bio-Engineering, Sam Higginbottom University of Agriculture, Technology and Sciences, Prayagraj, Uttar Pradesh 211007 India; 4 Department of Biotechnology, Siddharth University, Kapilvastu, Siddharth Nagar, 272202 Uttar Pradesh India; 5 Department of Molecular and Human Genetics, Banaras Hindu University, Varanasi, 221005 India

**Keywords:** AKT1, Point mutation, ADME, QSAR, Virtual docking, Dynamic simulations

## Abstract

**Background:**

Ovarian cancer is the world’s dreaded disease and its prevalence is expanding globally. The study of integrated molecular networks is crucial for the basic mechanism of cancer cells and their progression. During the present investigation, we have examined different flavonoids that target protein kinases B (AKT1) protein which exerts their anticancer efficiency intriguing the role in cross-talk cell signalling, by metabolic processes through *in-silico* approaches.

**Method:**

Molecular dynamics simulation (MDS) was performed to analyze and evaluate the stability of the complexes under physiological conditions and the results were congruent with molecular docking. This investigation revealed the effect of a point mutation (W80R), considered based on their frequency of occurrence, with AKT1 protein.

**Results:**

The ligand with high docking scores and favourable behaviour on dynamic simulations are proposed as potential W80R inhibitors. A virtual screening analysis was performed with 12,000 flavonoids satisfying Lipinski’s rule of five according to which drug-likeness is predicted based on its pharmacological and biological properties to be active and taken orally. The pharmacokinetic ADME (adsorption, digestion, metabolism, and excretion) studies featured drug-likeness. Subsequently, a statistically significant 3D-QSAR model of high correlation coefficient (R2) with 0.822 and cross-validation coefficient (Q2) with 0.6132 at 4 component PLS (partial least square) were used to verify the accuracy of the models. Taxifolin holds good interactions with the binding domain of W80R, highest Glide score of − 9.63 kcal/mol with OH of GLU^234^ and H bond ASP^274^ and LEU^156^ amino acid residues and one pi-cation interaction and one hydrophobic bond with LYS^276^.

**Conclusion:**

Natural compounds have always been a richest source of active compounds with a wide variety of structures, therefore, these compounds showed a special inspiration for medical chemists. The present study has aimed molecular docking and molecular dynamics simulation studies on taxifolin targeting W80R mutant protein of protein kinase B/serine- threonine kinase/AKT1 (EC:2.7.11.1) protein of ovarian cancer for designing therapeutic intervention. The expected result supported the molecular cause in a mutant form which resulted in a gain of ovarian cancer. Here we discussed validations computationally and yet experimental evaluation or in vivo studies are endorsed for further study. Several of these compounds should become the next marvels for early detection of ovarian cancer.

## Background

Ovarian cancer marks the most lethal gynaecological malignancy which ranks the fifth marveling cause of cancer deaths in females [1]**.** It is estimated that there are 22,530 cases with a mortality rate of approximately 13,980 deaths in the United States in 2019 [1] Ovarian cancers are categorized into 3 types based on cell origin: epithelial, stromal and germ cell [2]**.** The low survival rate and poor prognosis of ovarian cancer are due to a lack of screening methods at the early stages and ineffective treatments for advanced stages of disease [3]**.** Moreover it is very crucial to dissect the role of tumor-causing microenvironment during the early stage, proliferation, and metastasis**.** Thus, it becomes paramount to understand the root cause from different views of its molecular pathogenesis, histological subtypes, hereditary factors, epidemiology, methods of treatment, and diagnostic perspectives. The Cancer Genome Atlas (TCGA) revealed that the expression of AKT1, AKT2, and AKT3 was associated with poor patient survival [4]**.** The marveling cause of the disease is due to genetic and epigenetic changes of the cellular genome. Numerous small drug molecules of AKT gene targeting mutations such as FOXO, glucose metabolism (GSK3), and apoptotic proteins (BAD, NF-kB, FKHR) are available. Cell cycle arrest, apoptosis, DNA repair (MDM2) are critical in disease progression. Among various kinases, overexpression of AKT1 protein and associated mutations play a deciding role in cross-talk cell signalling in causing cancer. Recent studies have introduced assorted therapeutic agents as targets specific for cancer-driven factors involved in the inhibition of ovarian cancer development. One such factor of the kinase family is protein kinase B/serine-threonine (EC:2.7.11.1) (https://www.brenda-enzymes.org/index.php) serves as a decisive mediator of the P13K/AKT/mTOR cell signaling pathway that has distinct physiological functions such as cell growth, survival, proliferation, and metabolism [5]**.** Structurally AKT1 consists of three domains, including an N-terminal pleckstrin homology, a central catalytic kinases domain, and C-terminal domain [6].

AKT1 is the kinase that connects upstream signals from PI3K and mammalian targets of rapamycin complex2 (mTORC2) with downstream signals to mTORC1 and effectors such as mTOR, GSK3b along with phosphorylation cascade which acts as substrates that induce cell cycle progression, protein synthesis, lipid and protein phosphatases, glucose metabolism and cell growth [7]**.** AKT1 is mutated and AKT2 is amplified in about 40% AKT1 is inhibited by tumor suppressors including phosphatase and tensin homolog (PTEN) and inositol polyphosphate 4-phosphatase type 2 INPP4B [8, 9]. Therefore, targeting ATP binding cleft of AKT protein by inhibitors (natural/synthetic) has become an attractive strategy for treating patients in ovarian cancer. Interestingly, AKT1 protein inhibitors showed a strong binding affinity with mutant forms when compared to the native form. However, the emergence of acquired drug resistance in patients was found to limit its usage in the last phase of clinical trials. In ovarian cancer, overexpression of AKT is associated with advanced-stage platinum resistance [10, 11]. As an isoform of the AKT family, AKT1 is observed to be expressed unduly in a wide assortment of many human cancers including breast and ovarian cancers [12, 13]**.** The underlying molecular mechanism is assumed to cause conformational changes in native protein structure (AKT1) which modify covalent bond interaction by limiting their practical application. On that account, there is a need to search and develop novel as well as regimes that can counteract the drug resistance induced by the AKT1 gene. Even so, the molecular interactions and atomic stability for the W80R have also been determined for the present study**.**

W80R results in increased repression of FOXO 3 compared to wild type AKT1 in an invitro assay which is then predicted to result in a gain of AKT1 protein function. FOXO is a transcription factor in the nucleus that induces CGN2 transcription in epithelial ovarian cancer cells with enhanced catenin activity. The absence of Wnt ligand dissociates catenin from the destruction complex and translocates to the nucleus where it acts with the FOXO3 factor which is known to play a role in the W80R protein pathway. Abnormal activation of this pathway marvels to hyper-activation of catenin, which has been reported in ovarian cancers. W80R is one of the reported mutants of AKT1 cancer which cause missense driver mutation with 238 T > C of the coding sequence, also CDS (change in the nucleotide sequence as a result of mutation, where the syntax here used is identical to the method used for the peptide sequence) mutation c.238 T > A with gene location 14q32.33 [14] in the uterus section causing endometrial cancer. It has been proved that W80R contains highly conserved residues damaged by polyphen2, targeting through PI3K/AKT1/mTOR pathway of substitution-missense variant type affecting exon of protein domain PH (the UniProt Consortium 2019) and SIFT prediction as 3 [12]. The mutant W80R-Q79K on combination found to be displayed a very strong membrane localization and hyperactivation in transfected HeLa cells in both presence and absence of serum under fluorescence microscopy [15]. The previous studies of AKT1 co-occurring mutations (like Q79K-W80R) found to be hyperactive equal to E17K mutant widely distributed in different tissues such as endometrium (homozygous and heterozygous), large intestine (caecum), prostrate (with heterozygosity condition) breast cancers involving cross-talk signaling pathways [16]. The deleterious mutations of AKT1 (E17K and W80R) concluded to be of functional relevance exclusively in myxoid tumors [17]. The altering mutations promote growth factor independent cell proliferation as compared to wild type AKT [18]. AKT1gene alterations account for most of the genetic drive contributing to the pulmonary sclerosing haemangioma which is a benign tumor development [19]. It was observed in the patients receiving gnomically targeted therapy that W80R mutant found to be in clinical benefit of SD 4 mo + (stable disease), working efficiently with synthetic drugs temsirolimus and ixabepilone targeting ovary granulosa cell [20]. In line with, the inhibition of AKT1or its mutant proteins has been recognized as a compelling strategy for the treatment of cancers with [21] induce ovarian tumor angiogenesis [22] and in immune evasion [23].

Existing chemotherapeutic drugs have developed resistance to the novel compounds along with side effects despite enormous progress in anticancer drug discovery. Hence more targeted strategies are required to develop with sensitivity and specificity. Most of the successful anticancer compounds were originated from natural sources or as their analogs. Natural products and their synthetic analogues are a rich source of biologically active compounds which have been recognised as cancer stem cells (CSCs). These anti-CSCs natural products include flavinoids, stilbenes, quinines, terepenoids, polyketide antibiotics, steroids and alkaloids [24]. Flavonoids are naturally occurring secondary metabolites consisting of polyphenols having therapeutic benefits in multiple ways. These are low-molecular-weight compounds with non-nitrogenous properties consisting of C6-C3-C6 as a backbone with different classes [25] and their activities are structure-dependent. Chemically, flavonoids depend on their structural class, degree of hydroxylation, substitutions, and conjugations, and degree of polymerization [26]. Several mechanisms have been proposed for the effect of flavonoids at the initiation and promotion stages of the carcinogenicity including influences on development and hormonal activities [27]. Flavonoids fall under 6 different categories based on the functional group flavones (luteolin, apigenin), flavonols (quercetin, kaempferol), flavanones (naringenin), flavanonol (taxifolin), isoflavones, and flavan-3-ols (genistein, epicatechin, catechin, wedelactone, ellagic acid, silibinin, folstein, parthenoilods, oridonin, curcumin, reservertol. The choice of this study has relied on the compounds of the family called flavonoids with a tremendous variety of pharmacological and biochemical consequences including hepatoprotective, antidiabetic, cardio protective, anti-tumor, neuroprotective, and anti-inflammatory and played a wonderful role in the preclusion of Alzheimer’s disease**.** Equally studies on quercetin (QUR) demonstrated as its effect on anti-inflammatory, anti-apoptotic, antioxidant, and anticancer agent. This also found to improve the quality of oocytes and embryos. It affects the proliferation and apoptosis and thereby decreases in oxidative stress in granulose cells (GCs). Furthermore, it is also used as a complementary and alternative therapy in ovarian cancer with beneficial effects in treatment with PCOS (polycystic ovary syndrome) patient [28]. In an earlier investigation, this area has demanded series of chemical methods and animal models to synthesis marvel compounds with more time, investment, and level of exposure. To overcome this issue, computational approaches have opened doors for inquisition in predicting the mutation both in induced drug resistance and also to design resistance evading drugs. As a result of the above-mentioned shortfalls, the present study has aimed at the dynamic simulation at the molecular level and molecular docking studies on taxifolin targeting W80R mutant protein in protein kinase B/serine-threonine kinase/AKT1 protein of Ovarian cancer for designing therapeutic. This computational study relies on learning and pattern classification methods which can classify mutations create 3D protein structures.

## Materials and methods

### Sequence retrieval and structure analysis of selected protein

The amino acid sequence of AKT1 protein was retrieved from the Uniprot database with accession number P31749. The primary structure of the protein was elucidated using the ProtParam tool [29] of the Expasy server and the difference between physical and chemical properties of the AKT1 protein (wild) and mutant (W80R) were evaluated. Factors such as physicochemical properties, molecular weight, theoretical pI (isoelectric point), half-life, instability index (II), aliphatic index (AI), extinction coefficient (EI), grand average hydropathy (GRAVY), and site of origin were analyzed. The secondary structure properties prediction was carried out by the RAMPAGE server, which provides the configuration score like the total number of helices, turns, coils, predicted solvent accessibility, with the range, existed from 0 (highly buried) to 9 (exposed region) depending on the residue exposed. Normalized B-factor is measured for a selected protein as Z score which is a combination of template and profile-based prediction where residues are higher than zero are considered as less stable during experimental structures. The mutant protein W80R was edited manually at the amino acid position number and submitted to homology modelling [30].

### 3D modelling of W80R protein

Apart from successful experimental methods such as x-ray crystallography and nuclear magnetic resonance for 3D structures, there still exists the knowledge gap about the structural information about the protein. However, computational methodology fills the gap by an approach called Homology Modelling and makes it fit for the drug discovery purposes. The 480 amino acid residue length of W80R protein was retrieved to recognize the appropriate template sequence (PDB access code: 3O96) for structure modelling and functional prediction of the protein. This modelling depends mainly on a sequence alignment between the target and template sequence whose structure has been experimentally determined with [31, 32] the 3D structure of the target protein using its template was performed by MODELLAR (https://salilab.org/modeller/) and visualized by the PYMOL tool; based on template-target alignment. These theoretical structural models of the W80R protein were ranked based on the normalized discrete RMSD values. The model with the lowest RMSD score was considered as the best model [33].

### Evaluation of the structure model

The quality of AKT1 and mutant form W80R models were assessed by many tools to evaluate the stability and reliability of the model. PROCHECK suite [34] quantifies the residues in favorable zones of the Ramachandran plot, was used to evaluate the stereochemical quality of the model. ERRATA tool [35] finds the overall quality factor of the protein and was used to check the statistics of non-bonded interactions between different atom types. The compatibility of the atomic model (3D) with its amino acid sequence was determined using the VERIFY 3D program. Swiss PDB viewer 4.1.07 was used for the energy minimization of the predicted AKT1 protein along with its mutant form. The W80R model was further subjected to structural analysis and verification server to evaluate its quality, before and after energy minimization. ProSA tool [36] was employed for the refinement and validation of the minimized structure to check the native protein folding energy. The superimposition of the proposed model of AKT1 protein along with mutant form with its closest-structural homolog was carried out using chimera 1.11 [37].

### Selection and preparation of ligands

Natural compounds database containing more than 12,000 ligands were aimed to the AKT1 protein family were downloaded from the Pubchem database [38] and subjected to ligand preparation by ligprep wizard application of the Maestro 9.3 [39]. Ligprep tool was used to prepare the high quality of ligands, such as the addition of hydrogen’s, conversion of 2D to 3D structures, corrected bond angles and bond lengths, with lower energy structure, stereochemistry’s, and ring conformation followed by minimization in the optimized potential of OPLS 2005 force field [40, 41]. Properties such as ionization did not change and tautomers were not generated, specifically retained chiralities. Compounds were selected based on the lowest energy.

### Preparation of protein molecule and active site prediction

The W80R protein was modelled by using the protein preparation wizard of Schrodinger Suite; by adding hydrogen atoms, optimizing hydrogen bonds, and verifying the protonation states of His, Gln, and Asn. Energy minimization was carried out using constraint 0.3 Å RMSD and OPLS 2005 force field with steepest descent algorithm. The sitemap tool was used to identify binding pockets of W80R protein [42].

### Receptor grid generation

Receptor grid generation was done by the Glide application [42]. The receptor grid for W80R was generated using active site residues which were identified by Sitemap tool. Once the grid has been generated, the ligands are docked to the protein (W80R) using Glide version 5.8 (Grid-based Ligand Docking with Energetics) docking protocol. The scaling factor (0.25) and partial charge (1 Å) represent cut-offs of Vander Waals radius scaling.

### Molecular docking

Docking is the popular method of molecular modelling to build ligands into the active site of receptor molecule by estimating energy for the ligand binding to the protein [43, 44]. The value of this energy determines the biological activity of the molecules i.e. the higher energy, the more effective the drug based on the receptor will be considered. However, the term scoring (score) is used for calculation of binding energy by a ligand to a receptor molecule rather ranks assigned to position of ligand with their specific targets procedures were consistently carried out using a preparation of protein of Schrodinger and defining the grid on the active site of the protein. The reliability of the molecular docking is significantly affected by the accuracy of docking scores and the 3D structure of the receptor [45].

GLIDE **(Grid based ligand docking with energies)** molecular docking tool uses computational simulation methods for evaluating particular poses and ligand flexibility. GLIDE systematic method, a new approach for rapid, accurate molecular docking and its output G-score, is found to be an empirical scoring function, is a combination of diversified attributes. Glide uses the Emodel scoring function to select between protein-ligand complexes of a given ligand and the Glide Score function to rank-order compounds to separate compounds that bind strongly (actives) from those that don’t (inactives). G-score is calculated in Kcal/mol, encompass ligand-protein interaction energies, hydrophobic interactions, hydrogen bonds, internal energy, pi-pi stacking interactions, root mean square deviation (RMSD), and desolvation. GLIDE modules of the XP visualizes analysis of the specific ligand-protein interaction [46]. The ligands were docked using Extra Precision mode (XP) and conformers were evaluated using the Glide (G) score. The G score is calculated using this formula as:

$$ \boldsymbol{GScore}=\boldsymbol{a}\ast \boldsymbol{vdW}+\boldsymbol{b}\ast \boldsymbol{Coul}+\boldsymbol{Lipo}+\boldsymbol{Hbond}+\boldsymbol{Metal}+\boldsymbol{BuryP}+\boldsymbol{RotB}+\boldsymbol{Site} $$

where vdW denotes van der Waals energy, Coul denotes Columb energy, Lipo denotes lipophilic contact, H-bond indicates hydrogen bonding, Metal indicates metal-binding, BuryP indicates penalty for buried polar groups, RotB indicates penalty for freezing rotatable bonds, site denotes polar interactions in the active site and a = 0.065 while b = 0.130 were the coefficients of vdW and Coul.

**ADME properties studies** Calculation of absorption, distribution, metabolism, excretion, and toxicity (ADME/T) properties was performed for best-docked ligand molecules by QikProp software. This software predicts various limiting factors such as QP log Po/w, QPlog BB, SASA, FOSA, FISA, PISA, WPSA, volume, donarHB, acceptorHB, dip^2/V, AC*DN*5, Caco, QlogS, rotors, rule of 5, rule of 3, the overall percentage of human oral absorption, etc. [47]. Lipinski’s rule of five [48] measures the drug-likeness for the prediction of a chemical compound as an orally active drug based on biological compounds and pharmacological properties.

### Analysis of cancer-associated mutants

The deleterious W80R mutations that are specific for cancers were predicted using the FATHMM server **(**http://fathmm.biocompute.org.uk/**)** **[**49**]** which allows the distinct difference between cancer-promoting/driver mutations and other germline polymorphisms. The gene number identifiers (UniProt id) along with mutant form as a text were provided as the input for the prediction.

### Molecular alignment and 3D QSAR studies and validation

The key component of 3D QSAR analysis is the arrangement of the molecules based on the scaffold they share which generated using the training was set of 44 molecular poses with a grid spacing of 1 Å PLS (partial least square) algorithm to establish the relationship between biological activity and different structural features. The training set was adjusted to 50%. Three models were generated by Gaussian filed extension as Gaussian steric, electrostatic, hydrophobic, hydrogen bond donor, hydrogen bond acceptor, and aromatic ring fields. CoMFA and CoMSIA are the tools employed as independent variables in PLS regression analysis. The best model was chosen based on the criteria of statistical robustness and visualized using contour map modules. The predictive power and stable models were assessed using the leave one odd (LOO) cross-validation method. The crucial aspects for the test set statistics include RMSE, Q2, SD, R^2^, R^2^CV, R^2^scramble, stability, F, P, Q2, Pearson’s r which indicates the predictive ability of the model. A Scatter plot was generated in correlation with predicted activity on the Y-axis and observed activity on the X-axis of the data set model [50].

### Contour maps visualisation

Representation of the fields as contours (surfaces) or as color intensities of the fields on the grid can be displayed in different styles. Based on the field type, the colors are designed and field intensities are shown for one field at a time. The fields with greater absolute values than the cut-off were presented at the maximum brightness.

### Molecular dynamics simulation

The simulation of protein-ligand complexes was implemented by GROMACS 4.5.5(Groningen machine for Chemical Simulations) software [51]**.** The complex with the lowest binding energy was selected for molecular dynamics (MD) simulation. The ligand parameters were analyzed using PRODRG online server [52] in the framework of GROMACS force-field 43a. The ligand enzyme complex was solvated at a simple point charge as well as a water box under periodic boundary conditions using 1.0 nm distance protein to the box faces. The system was then neutralized by Cl^−^ or Na^+^ counter ions for the W80R complex with ligand respectively. To perform energy minimization, the complex was equilibrated under volume, constant number of particles, and temperature condition for 100 ps at 300 k, followed by 100 ps. All the covalent bonds with hydrogen bonds were considered using a linear constraint solver algorithm. The electrostatic interactions were treated using the particle mesh Ewald method [53]**.** Further MD simulation studies were noted for 20 ns to check the accuracy and stability of the ligand-protein complexes. The potential of each trajectory produced after MD simulations were analyzed using g_rms, g_rmsf, and g_h bond of GROMACS utilities [54] the root mean square deviation (RMSD), the root mean square fluctuation (RMSF), with hydrogen bonds formed between the ligand and protein complex.

## Results

### Mutant W80R sequence analysis

The development of anticancer compounds with variegated pharmacological effects becomes a very paramount topic and hence main class of secondary metabolites, both dietary and synthetic flavonoids have been subjected to clinical trials [55]**.** Definite beneficial biological activities of dietary flavonoids including antioxidants [56] anticancer [57], and cardio-protective properties [58] have been identified in a series of previous studies. Flavonoids are known for their wide exposure to chemo-preventive, chemotherapeutic activities, and the availability of the compound in plant sources for the human diet in routine consumption [59].

The analysis of the mutant W80R protein sequence of the AKT1 has 480 amino acid residue which plays a very crucial role in metabolism, cell proliferation, cell survival, growth, and angiogenesis, was downloaded from Uniprot with accession number (P31750). The amino acids in the protein sequence of W80R were composed of lysine, leucine, glutamic acid, and alanine. The ProtParam tool was used for the W80R protein sequence to compute physio-chemical parameters such as molecular weight of 5565.45 kD. The W80R had a pI (isoelectric point) of 5.99 indicating its acidic nature (pI< 7.0) with an aliphatic index (AI) (71.69). The protein volume is occupied by aliphatic side chains such as lysine, leucine, glutamic acid, and alanine. The instability index of W80R measured 35.76 of the unstable nature. The grand average of hydropathicity (GRAVY) of W80R protein was lower (− 0.583), which proves its high affinity with water. The comparison of statistical characteristics is showing the differences among wild AKT1 and mutant W80R using the ProtParam tool (Table 1). The comparison of sequence analysis of W80R mutant protein with AKT1(wild) at nucleotide and protein level was same with a slight difference, thus proving-T, C-G rich region, and properties such as molecular weight, amino acid composition, theoretical pI, aliphatic index, and grand average of hydropathicity (GRAVY) were found in an appropriate range of influencing the protein stability.

**Table 1:** Comparison of primary sequence analysis using the ProtParam tool between AKT1 (wild) and W80R (mutant)

S.No	Parameters	AKT1	W80R
1	Molecular weight	5586.7kD	5565.4kD
2	pI	5.75	5.99
3	Aliphatic Index	71.69	71.69
4	Instability Index	35.47	35.76
5	GRAVY	−0.575	−0.583
6	Atoms	7772	7776
7	Total number of Asp+Glu residues in a protein content	77	76
8	Total number of Arg + Lysresidues in a protein content	66	68

### 3D molecular modelling of W80R mutant protein

The 480 amino acid residue length of W80R protein was subjected to BLASTp analysis against RCSB PDB to identify the suitable template for comparative structural modelling and functional prediction. The result of the BLASTp search revealed a template (PDB id 3O96) of high-level identity with the target sequence of AKT1. The query coverage (100%) showed a high degree of identity between two proteins (AKT1 and W80R) of 480 sequence length, and E value (2e-60) is the expected value obtained by hits, percentage identity defines the extent of two sequences, Modeller 9.13 has generated 5 models of W80R, among these the lowest score is considered as stable which is thermodynamically subjected to further refinement. The lowest RMSD as 0.18 score model was considered as the best one for further validation purposes [33]**.** Finally, three dimensional (3D) structure of the selected protein using its template was visualized by the PYMOL tool.

### Model assessment and validation

**Fig. 1:**
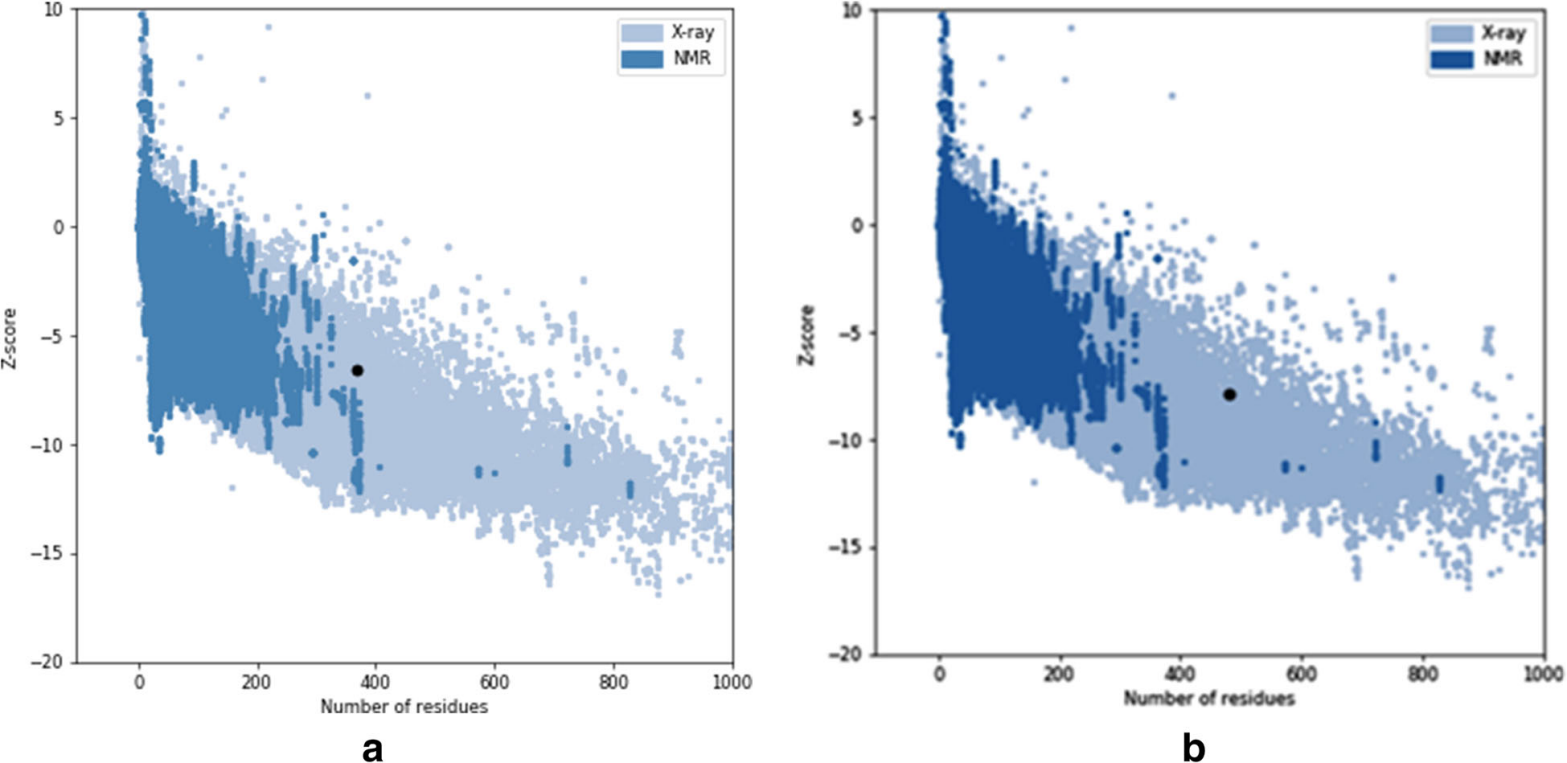
Protein structure analysis (ProSA) of the W80R (mutant) on the left side and AKT1(wild) on the right side. (**A**) The overall quality of the W80R model represents a Z score of −7.9Kcal/mol (**B**) Overall quality of the wild protein AKT1 represents a Z score of − 7.2Kcal/mol

The stability of the protein was constructed based on the backbone of torsion angles psi and phi which were evaluated by the PROCHECK server that computes the amino acid residues in the existing zones of Ramachandran plot analysis of W80R mutant forms **(**Table 2**).** The information presented in Table 2 depicts the Ramachandran plot through RAMPAGE server where W80R mutant protein has 79.3% amino acids falls in the most favored region with located major active binding sites, while 13.8% in an allowed region and 6.9% residues in the outlier region of the plot with lesser significance. SAVES analysis was conducted to confirm the quality of the protein model followed by ProSA, RMSD assessment for a high-quality structural model for virtual screening. The quality of the predicted model of AKT1 protein and a W80R mutant was supported by a high ERRAT score of 81.99 in an acceptable protein environment. The VERIFY 3D results of W80R showed 81.88% of the residues with an average 3D-1D score > = 0.2, indicating the stability of the model. ‘WHAT IF’ tool examines the coarse packing quality, the model protein structure, reflecting the acceptance of good quality**.** The reliability of the W80R form was confirmed by ProSA **(**Fig. 1**)** which achieved a Z score of − 7.92 kcal/mol compared to the wild form AKT1 having a Z score − 7.2 kcal/mol, wherein the energy is negative, reflects the best quality of the model. The quality of the model was evaluated through the comparison of predicted structure with experimentally determined structure followed by superimposition and atoms RMSD assessment using Chimera 1.11, which proved that the predicted model is good and quite similar to the wild protein.

**Table 2:** Comparison of secondary structure using RAMPAGE server between AKT1and W80R mutants

S.No	Protein Properties	AKT1(wild)	W80R(mutant)
1	Total amino acids	480	480
2	Number of residues in favoured region (−98.0% expected)	388 (81.2%)	379 (79.3%)
	Number of residues in allowed region(−2.0% expected)	61 (12.8%)	66 (13.8%)
3	Number of residues in outlier region	29 (6.1%)	33 (6.9%)

### Active site and score prediction

A proven algorithm for binding site identification and evaluation of the drug ability of those sites marvels to modify hit-compounds to enhance receptor complementarities. The active site was performed using a sitemap tool to assess each site by calculating attributes such as size, volume, amino acid exposure, hydrophobicity, hydrophilicity, donor/acceptor ratio. The most reliable score was obtained in the binding pockets of W80R. The predicted amino acids in the active region were LEU^156^, GLY^157^, GLU^234^, MET^281^, ASN^279^, GLU^278^, LYS^276^, ASP^274^, THR^291^, ASP^292^, PHE^293^, GLY^294^, LEU^295^, GLU^298^of site score for the selected model was 1.128, drug ability score − 1.149 with Volume 384.486 and size measured was 179 for further docking analysis.

### Analysis of cancer-associated mutants

The mutation impact for the protein W80R was classified using the FATHMM server derived from the new FATHMM-MKL algorithm. It distinguishes between cancer-promoting/driver mutations and other germline polymorphisms. This algorithm predicts the functional, molecular, and phenotypic consequences of the missense mutation of a functional protein using hidden Markov models (HMMs), representing the alignment of homologous sequences and conserved protein domains with “pathogenicity weights”, representing overall tolerance of protein/domain to mutations [49]**.** The gene number identifier (UniProt id) along with mutant form as a text was provided as the input for the prediction based on the FATHMM server predictions with a score − 1.12 responsible for benign cancer. The functional scores for individual mutations were obtained from the FATHMM-MKL server which falls in the range of 0–1 known as single p- values fall in the range of (0–1) where the values below 0.5 are predicted as benign and above 0.5 are deleterious.

### Determination of ADME profile

Molecular properties of the selected compounds were studied using Qikprop and chosen based on the Lipinski rule of five which marks the most important activity in drug discovery and development. Multifarious Insilco techniques have been employed to measure the drug-likeness for a compound based on numerous descriptors. Calculation of absorption, distribution, metabolism, excretion, and toxicity (ADME/T) properties was predicted for best-docked ligand molecules using Qikprop software. Qikprop computes almost 20 physical descriptors over a wide range of predicted properties unlike a fragment-based approach, by screening compound libraries for hits and play a marvel optimization that can be used to improve predictions by fitting to experimental data and also to generate QSAR models. The detailed analyses of chemical and molecular descriptors and also solubility properties were tabulated in Table 3, 4, and 5. The results of ADME properties are an important index to check the clinical candidates have reached the required standard. It is revealed that compounds in the table were ranked based on the potential drug properties. According to a previous study, ~ 40% of failures to develop medicine in the development phase are due to poor biopharmaceutical properties (pKa-dissociation constant and bioavailability) [60]**.** The ADME as a deal medicine has the following characteristics,hydrogen bond donar< 5; hydrogen bond acceptor < 10; molecular weight < 500 Da;lipid water partition coefficient < 5; water solubility partition coefficient − 6.5 < logs< 0.5; and polar surface area 7.0–20.

**Table 3:**
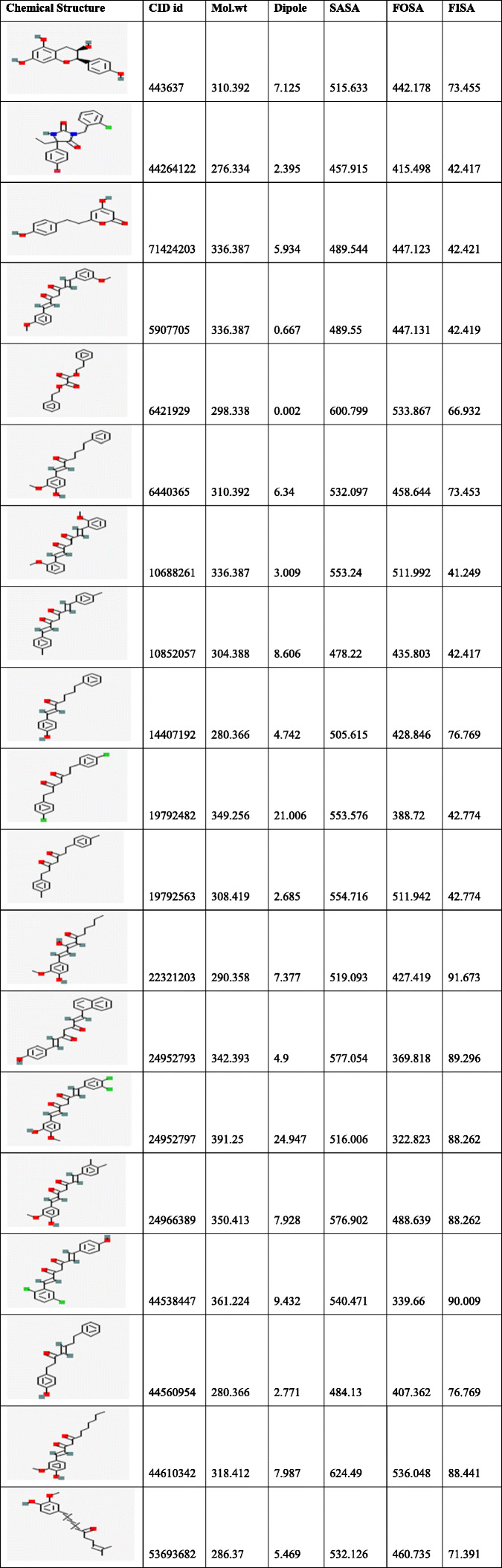
Detailed analysis of ADME properties of ligands using QIKPROP software

*SASA: total solvent accessible surface area in square angstroms using a probe with a 1.4A radius; **FISA:** hydrophilic component of the SASA (SASA on N, O, and H on heteroatom); **FOSA:** hydrophobic component of the SASA (saturated carbon and attached hydrogen); **CID ID:** compound Id from PubChem database.

**Table 4:**
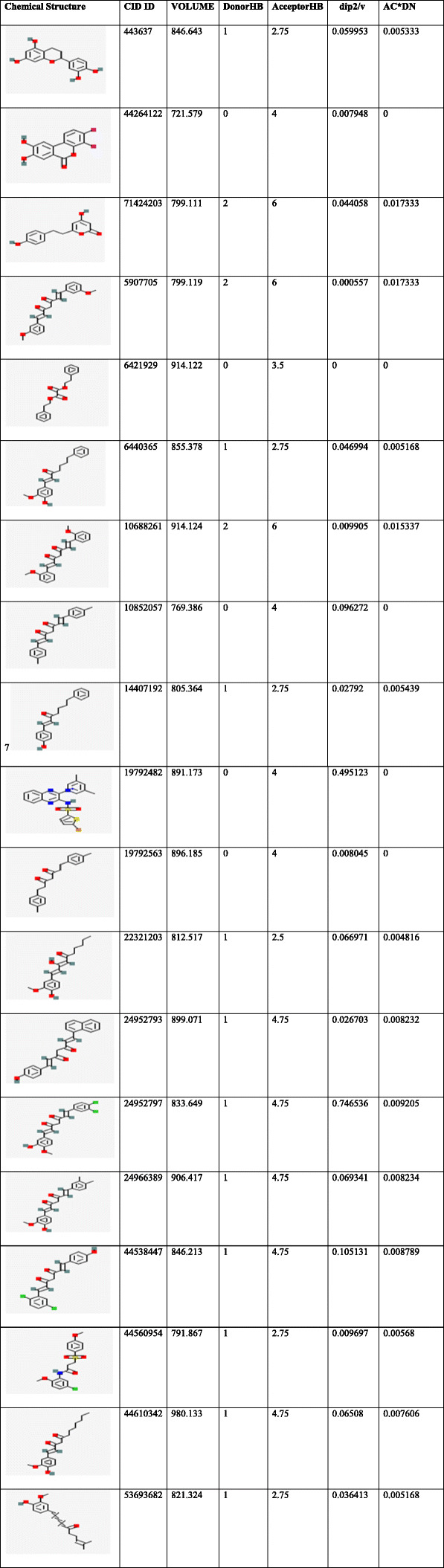
Detailed analysis of ADME properties of ligands using QIKPROP software

*.**Donor HB:** it is the calculated number of hydrogen bonds that would be donated by the solute to water molecules in an aqueous solution, values are averages take over many configurations, so they can be non-integer; **Acceptor HB:** it is estimated as the number of hydrogen bonds that would be accepted by the solute from water molecules in aqueous solution; **dip2/v:** square of the dipole moment divided by the molecular volume. This is the key term given in Kirkwood-Onsager equation for the free energy described of solvation of a dipole moment with volume V; AC*DN: index of cohesive interaction in solids; **Volume:** total solvent-accessible volume in the cubic angstroms using a probe with 1.4 A radius.

**Table 5:** Solubility prediction parameters for molecular descriptors

CID	QPlogpC1	QPlogPW	Qplogpoct	QPlogPw	QPlogPo	QPlogS	ClQPlogS	QPlogHER	QPPCaco	QPPMDCK	QPlogKp
5,318,278.0	27.1	6.7	11.8	5.4	2.9	−4.5	−3.0	− 4.1	1992.1	1042.1	− 2.8
5,469,422.0	22.7	5.0	8.7	5.1	1.8	−2.8	−1.9	− 4.0	3923.3	2167.8	−2.3
5,469,423.0	25.8	6.3	14.3	10.0	1.5	−3.3	−2.2	− 3.9	3923.0	2167.6	−2.3
5,907,705.0	25.8	6.3	13.7	10.0	1.5	−3.3	−2.2	−3.9	3923.1	2167.7	−2.3
443,637.0	30.4	6.9	10.9	5.1	3.1	−5.8	−2.6	−5.4	2297.1	1215.5	−2.8
44,264,122.0	27.4	6.7	11.7	5.5	3.0	−4.8	− 3.0	− 4.5	1992.2	1042.1	−2.8
71,424,203.0	30.4	7.3	15.4	10.2	2.1	−4.3	− 2.2	− 4.4	4024.7	2228.4	−2.3
10,852,057.0	24.6	5.5	10.6	5.1	2.1	−3.2	−2.3	−4.0	3923.3	2167.8	−2.3
14,407,192.0	25.4	6.2	10.8	5.4	2.6	−4.3	−2.6	−4.3	1853.1	963.6	−2.8
19,792,482.0	29.5	7.5	18.3	5.3	3.3	−5.1	− 3.4	− 4.6	3892.8	10,000.0	−2.3
19,792,563.0	29.7	6.6	11.1	5.3	2.9	−4.6	−2.4	− 4.6	3892.8	2149.6	−2.3
22,321,203.0	25.1	6.3	11.1	5.1	2.7	−4.5	−2.9	−4.6	1338.3	677.9	−3.0
24,952,793.0	30.3	−7.7	13.7	8.4	2.4	−4.9	−3.3	−18.3	1409.6	717.0	−2.7
24,952,797.0	26.6	−9.3	22.7	7.5	2.3	−4.3	−3.9	−17.7	1441.8	2759.8	−3.1
24,966,389.0	29.5	−8.5	13.8	7.6	2.4	−4.9	−2.9	−17.7	1441.8	734.7	−3.1
44,538,447.0	27.1	−8.6	14.0	7.6	2.4	−4.8	−3.4	−17.7	1387.8	2852.3	−3.1
44,560,954.0	24.9	−10.9	10.4	5.4	2.5	−4.0	−2.6	−17.7	1853.1	963.7	−2.8
44,610,342.0	32.4	−7.3	14.7	7.7	2.8	−5.8	−2.5	−17.7	1436.1	731.6	−3.1
53,693,682.0	26.1	−9.8	11.0	5.4	2.7	−4.8	−2.7	−17.7	2084.0	1094.1	−2.739

*QPlogPoct: predictedoctanol/gas partition coefficient;**QPlogPw:**predicted water/gas partition coefficient;**QPlogPo/w**: predicted octanol/water partition coefficient;**ClQPlogS:** conformation –independent predicted aqueous solubility, logs. S in mol dm^− 3^ is the concentration of the solute in a saturated solution that is in equilibrium with the crystalline solid; **QPlogHERG**: predicted IC 50value for blockage of HERG K^+^ channels;**QPPCaCo:** predicted apparent CaCo-2 cell permeability in nm/sec; Caco-2 cells are a model for the gut blood barrier;**QPlogKp**: predicted skin permeability, logKp; **QPlogS:** Predicted aqueous solubility, log S, S in mol dm^− 3^ is the concentration of solute in the saturated solution that is in equilibrium with the crystalline solid; **QPPMDCK:** Predicted apparent MDCK cell permeability in nm/sec, MDCK cells are considered to be a good mimic for the blood- barrier;**QPlogpCl**:Predicted hexadecane/gas partition coefficient

### QSAR studies and validation

**Fig. 2:**
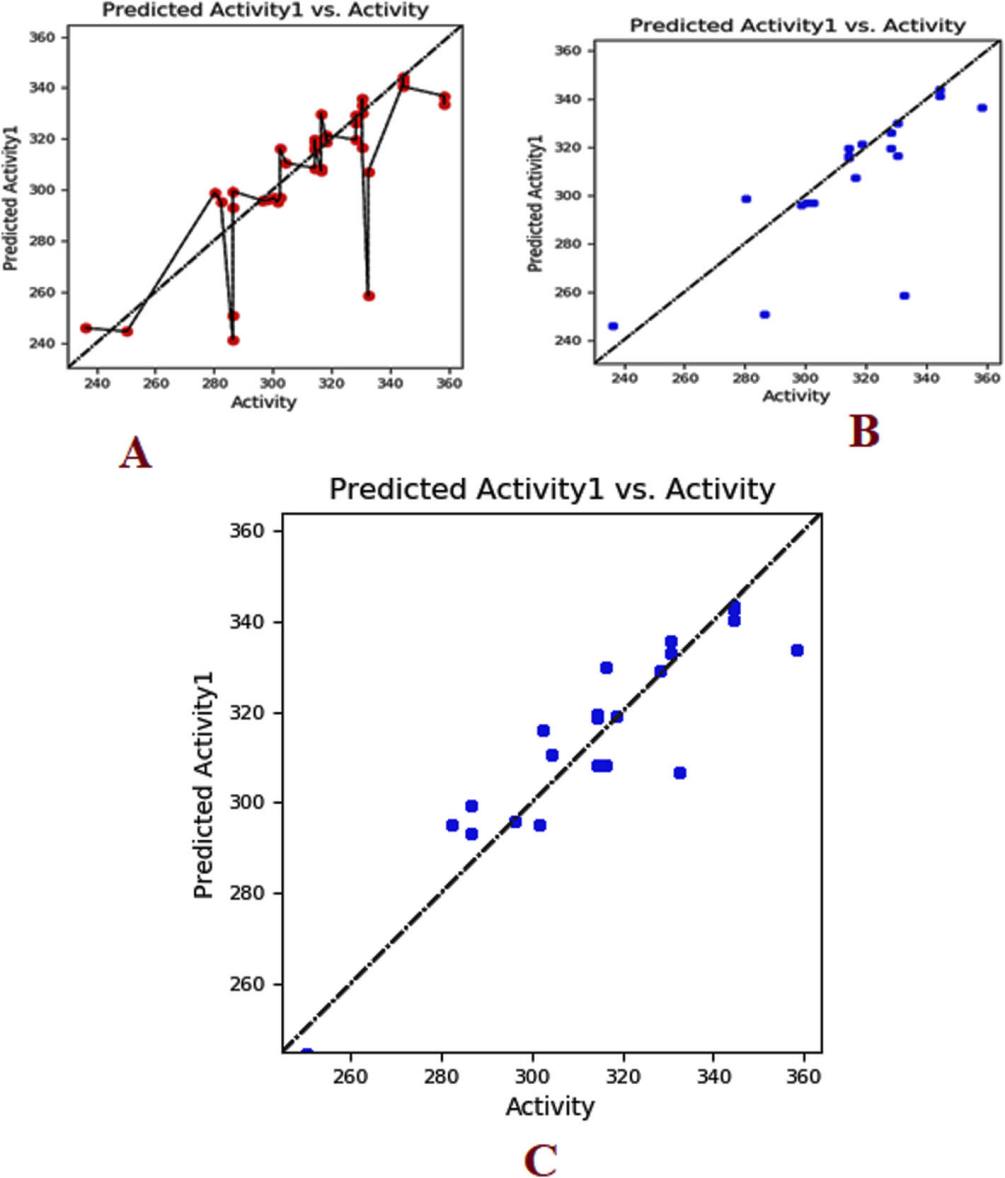
(**A**) Scatter plot diagram between actual activity and predicted activity showing QSAR results of all molecular descriptors (**B**) Activity predicted between only training set chemical descriptors(**C**) Predicted activity between test set of chemical descriptor

A dataset of 44 ligand compounds was chosen for statistical studies and classified as the training set and test set into 50% for suitable 3D QSAR model development. The graphical interface allowed building dataset into training and testing equally for 50% by generating a correlation coefficient. The graph obtained for all/training models/test models was observed in Fig. 2A. Molecular descriptors (ligands) were divided into a training set and test set (Table 6) with parameters such as phase QSAR, phase activity, % extrapolation, predicted error and predicted activity. QSAR built model was generated based on docking poses and substructure alignment was represented with standard deviation for the regression distributed over n-m-1 degrees of freedom(n ligands, m PLS factors) as 10.7913, R^2^(the coefficient of determination) gives 0.8226 means the model accounts for 82% of the variance in the observed activity, which falls between 0 and 1, R^2^C yields 0.2055 for cross-validated where R^2^ is obtained by leaving an N-out approach, R^2^scramble (R^2^ is regression or coefficient of determination) obtained as 0.4889 which computes the average value obtained using scrambled activities of Fig. 2B. This parameter is calculated from a series of models built using scrambled activities. The lower values indicate that the model cannot fit for random data while higher the value means that the variable set can be fairly complete and allows fitting anything. R^2^ value indicates the data set is over fit. Larger the value, the larger the data occupancy. The stability provides the degree for molecular fields that can fit random data, however, statistics observed to be 0.379 for the model prediction of the changes obtained in the training set composition. The descriptor F gives the ratio of the model variance to the observed activity variance. The model variance is calculated for the distributed data over m degrees of freedom and the activity variance is distributed over n-m-1 (n ligands, m PLS factors) hence obtained value of 92.7 means indicates more significant regression. Pearson descriptor measured 5.95e-09, where the significance level treated as a ratio of Chi-squared distributions. The smaller values indicate a greater degree of confidence while a *P* value of 0.05 means F is significant at a 95.6% level. RMSE- root mean square error predictions for the test set were to be 22.02, Q^^2^for for predicted activities with 0.2915. If the value becomes negative, then the variance in the errors is larger than the variance from the observed activity. Pearson-r correlated with predicted activity, and observed activity observed for test set with 0.7508. The test set was determined within the maximum range of the training set. The field values for the ligand were estimated outside the range found in the training set in percentages was calculated under Extrapolation of the complete data set.

**Table 6:**
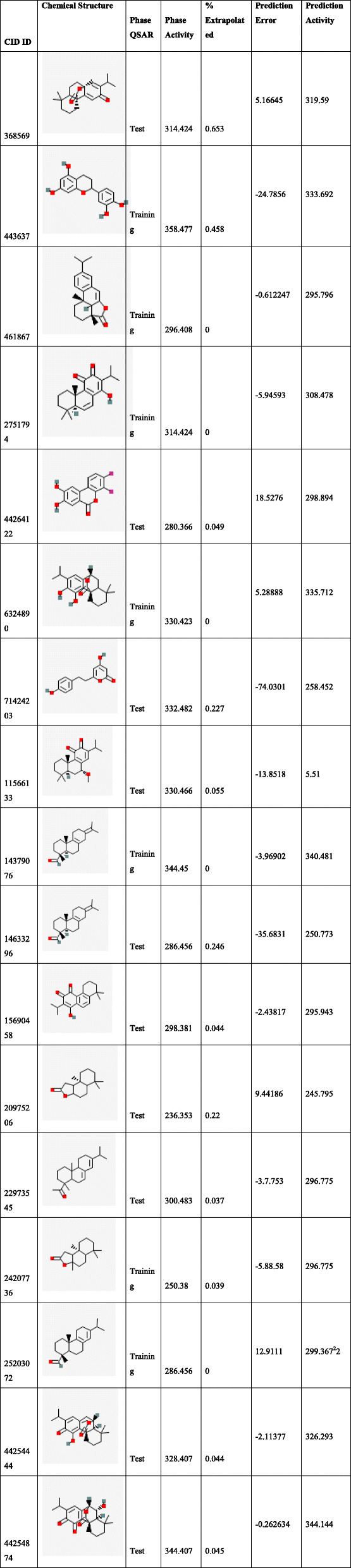
List of statistical analysis generated using field-based QSAR

### Contour visualisation

**Fig. 3:**
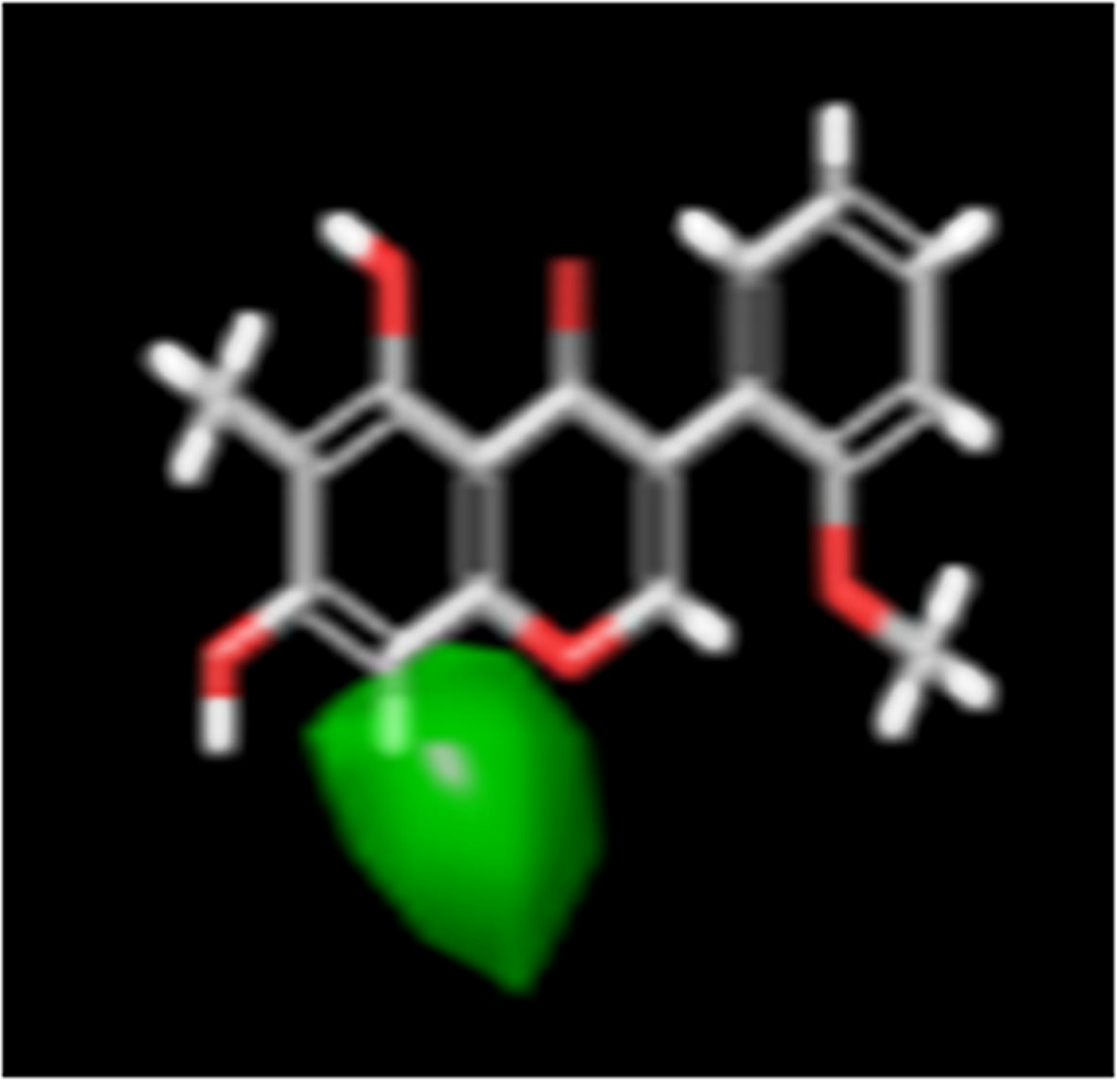
The CoMFA steric field with 2 Å grid spacing is displayed for the compound CID: 5482167, which depicts hydrophobicity via contour mapping and demonstrates the C7 substituted group was in the solvent accessible hydrophobic pocket. Steric fields are considered as most favorable regions with high glide score

The contour maps (Fig. 3) were used to illustrate the fields required for biological activity. Field-based QSAR interface creates electrostatic, hydrophobic, and steric fields for optimization and marvels discovery. The represented green contour indicates the bulky group in a favourable region. The contour map depicts hydrophobicity in the solvent-accessible hydrophobic pocket steric fields are considered as the most favourable regions with a high Glide score. The obtained results have shown the steric and Gaussian field fractions are much larger than other fields suggesting most of the binding energy has been contributed from hydrophobic interactions.

### Molecular docking studies

**Fig. 4:**
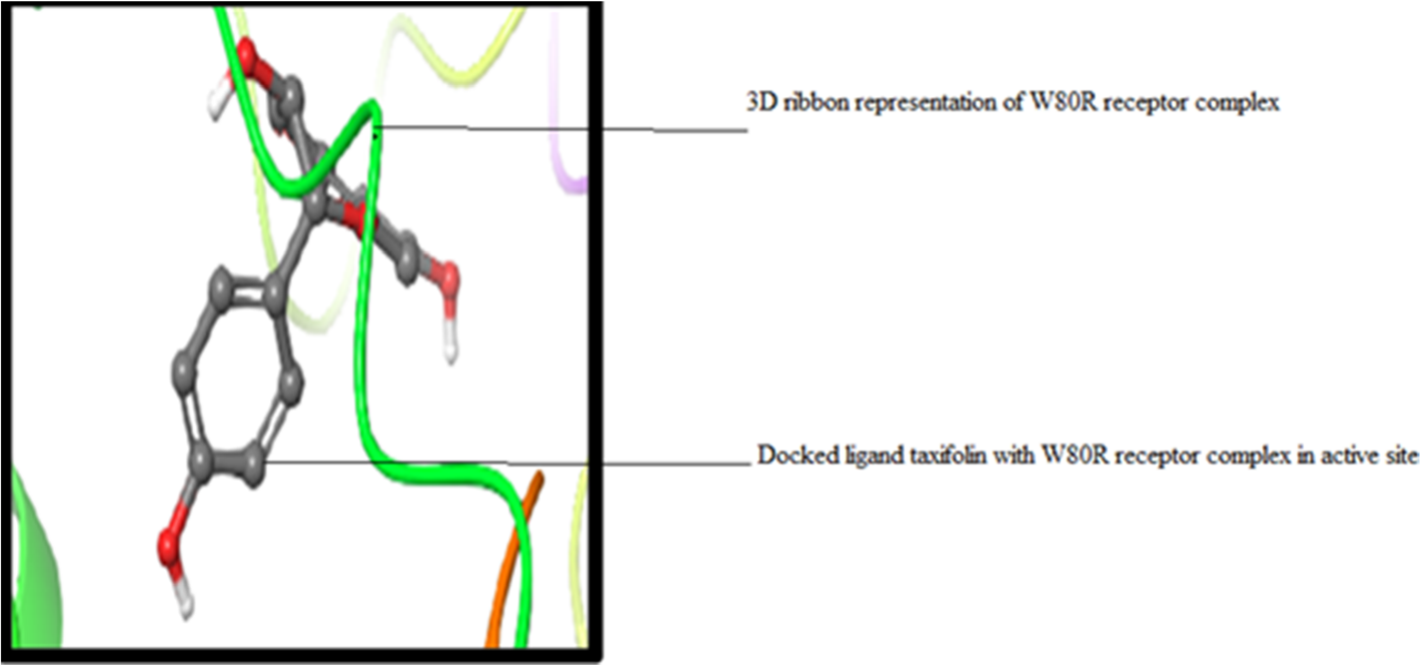
A docked complex of W80R protein in ribbon model with inhibitor CID ID 443637 (Taxifolin) at an active site of binding pocket with XP score − 9.63 kcal/mol

Molecular docking is the paramount computational tool to configure (Fig. 4) all the possible active conformations of binding at the active site for the receptor molecule. Before performing the docking protocol, the co-crystallized ligand was re-docked into the crystal structure of the 4GV1 (AKT1) receptor molecule to evaluate the reliability of the standard precision algorithm of the Glide. A dataset of flavonoids family along with its structural analogs comprising 7000 ligands was selected. Upon generation of Epik for suitable tautomeric states per 16 for each ligand, 12,000 ligands were chosen entirely as a whole set for virtual screening with W80R mutant protein. The top three ligands with the best binding energy were considered for further analysis (Fig. 4).

The major contribution is incorporation of interaction energies of Couloumb and vdW between ligand and the receptor. The wide disparities from the original interaction energies seem to be reduced greatly, although charge- charge interactions were found to be favoured to an extent. The coloumb-vdW energies used in Glide score 2.5 employ these reductions in net charge except in anionic ligand-metal interactions, for which glide uses the full interaction energy.

**Fig. 5:**
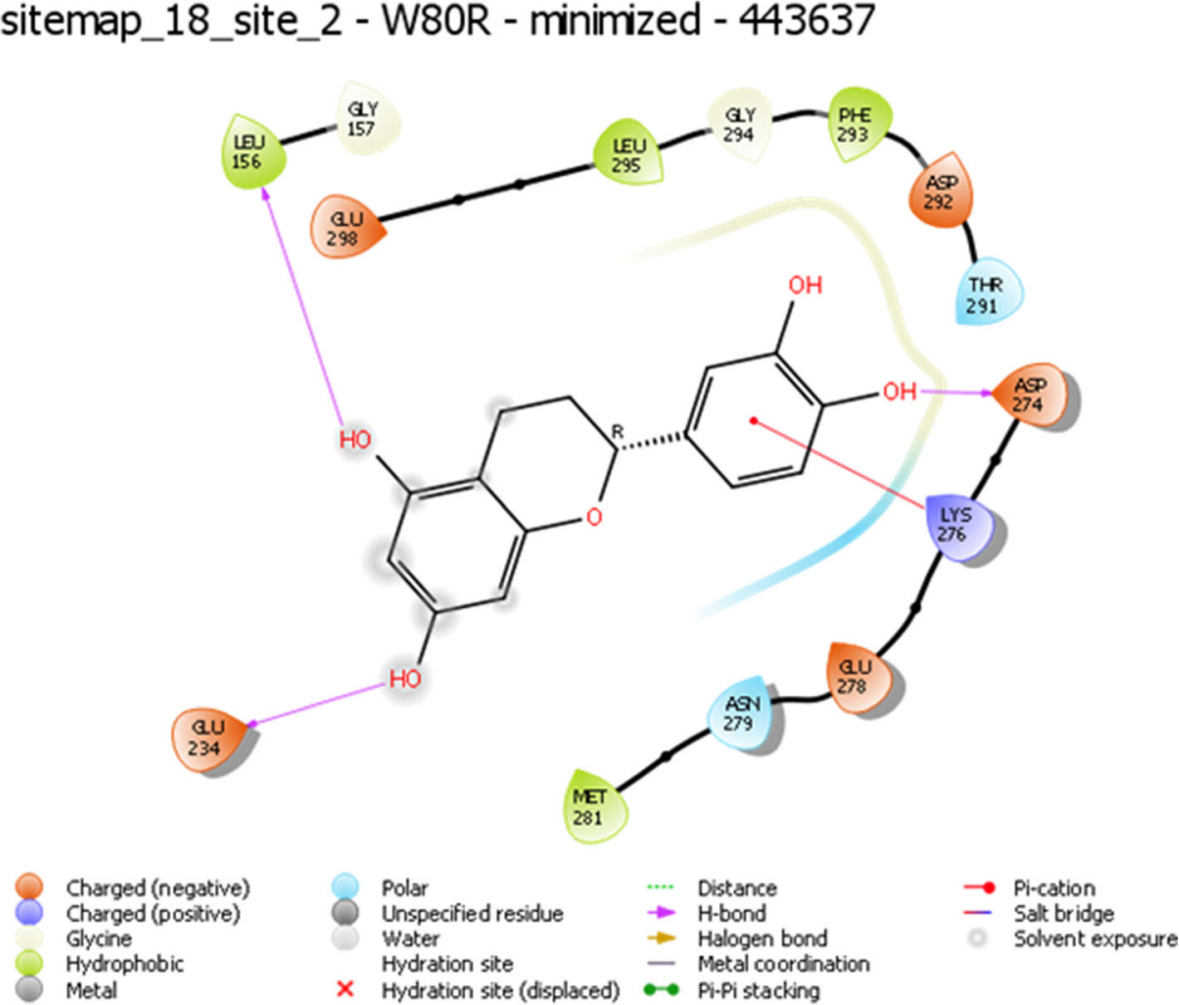
Representation of W80R receptor molecule with CID ID-443637 as a ligand interaction with protein residues LEU^156^, GLU^234^, and ASP^274^ of hydroxyl group (−OH) and 1 pi-cation interaction with LYS^276^ with noticeable solvent exposure sites observed at some residue locations with highest Glide XP score of −9.63Kcal/ mol

**Fig. 6:**
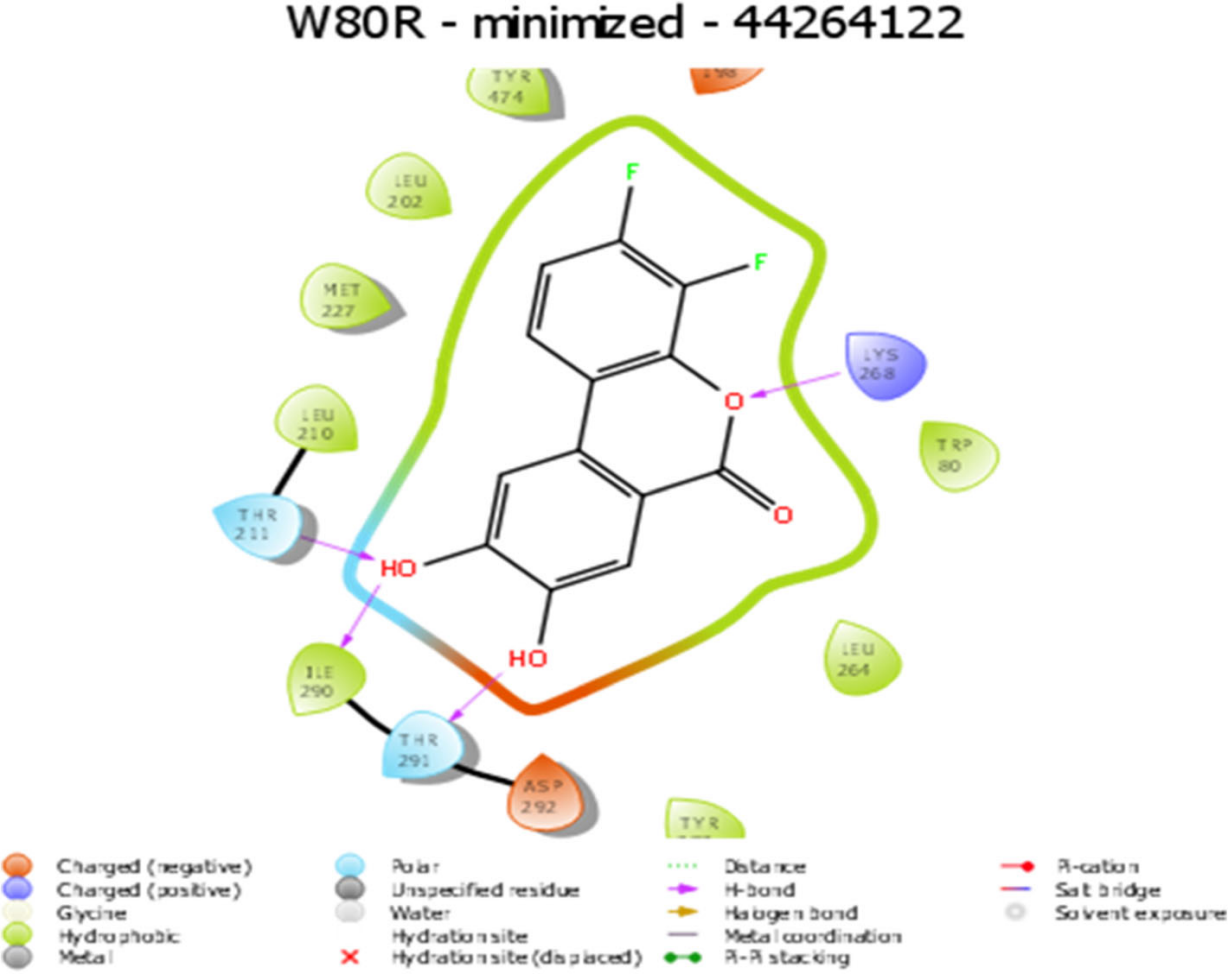
Representation of W80R protein-inhibitor complex of CID 44264122 with three hydrogen bonds of a hydroxyl group (−OH) interacting with THR^211^, ILE^290^, and THR^291^ and oxy bond with residue LYS^268^ with Glide XP score: −9.43Kcal/mol

**Fig. 7:**
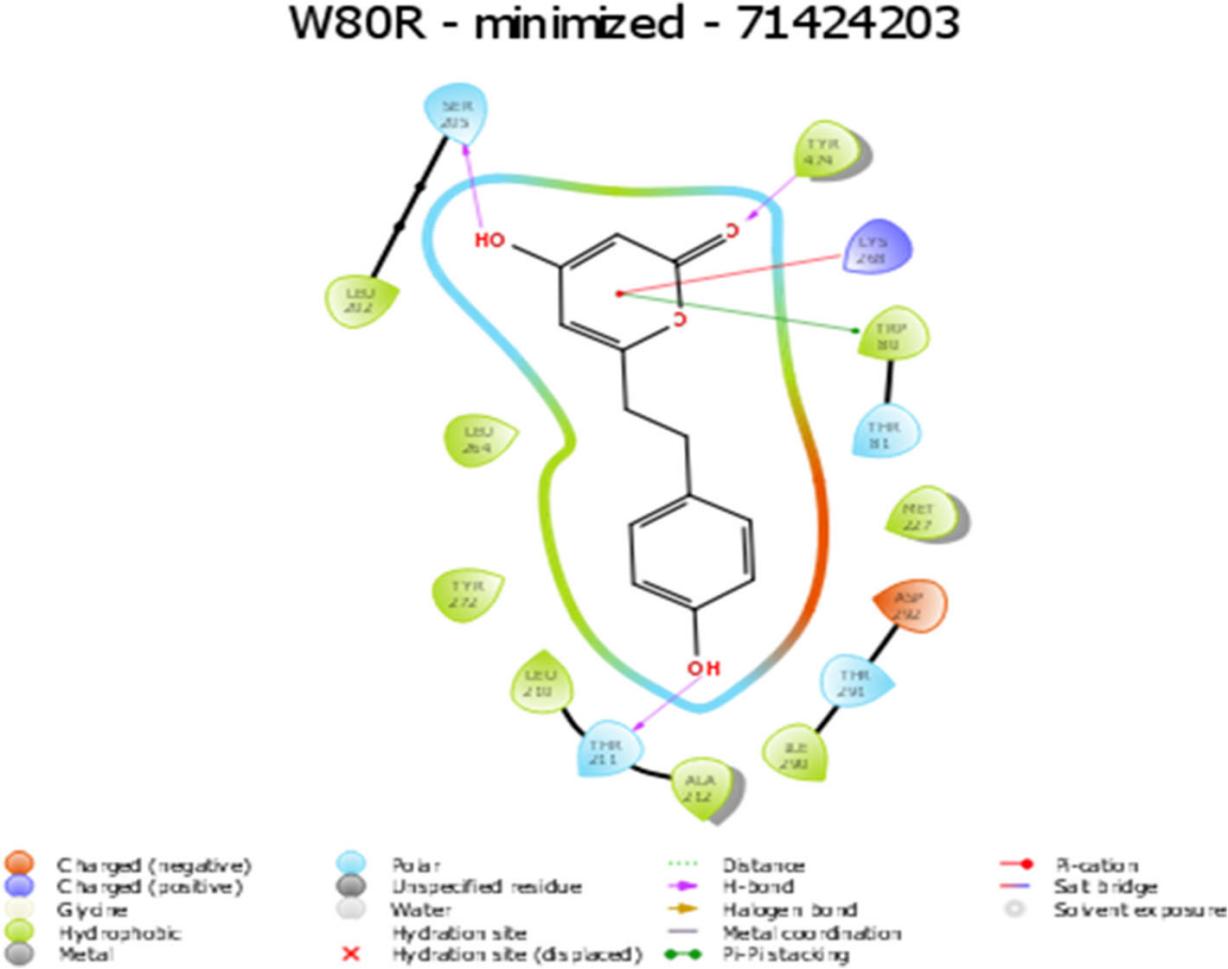
Representation of W80R receptor molecule with inhibitor at the active site showing protein-ligand hydrogen bond interaction with residues as TYR^474^, SER^215,^ THR^211^, with 1 pi-pi interaction at TRP^80^ residue, and 1 pi-cationic interaction bonding with LYS^265^ with G score − 9.36Kcal/mol

Several hydrogen bond interactions were found in the docking result. The top-scoring compound belongs to CID-443637 was having lower binding energy with a Glide score of − 9.63 Kcal/mol. The hydroxyl group of LEU^156^, GLU^234^ and ASP^274^ forms hydrogen bond revealing the strongest stability with the receptor molecule. The three hydrogen bond interactions provide the guarantee for stable conformation of a binding ligand molecule to protein structure which influences the activity of the ligand. The interaction with 1 pi ~ cation LYS^268^ recognized as energetically significant [61] and noncovalent binding interaction proves to exist in a quite strong platform both in the gas phase and liquid media [62] which is a special hydrophobic interaction having a cationic side chain amino acid, indicating that the geometry is biased towards aromatic amino acid, one that experiences a favorable pi~ cation interaction [63] having IUPAC name 2-(3,4-dihydroxy phenyl)-3,4 –dihydro-2H-chromene-5,7-diol of C15H14O5 (Fig. 5). The second highest molecule of 44264122 has a binding energy of -9.43 kcal/mol with hydrophobic contacts with residues such as THR^291^, ILE^290^, THR^211^ and oxy bond with LYS^268^ having IUPAC name 3,4 Difluoro-8,9-dihydroxbenzo[c] chromen-6-one of C13H6F2O4 (Fig. 6). The third compound CID 71424203 has the binding energy of − 9.36 kcal/mol forming hydrogen bond interactions with amino acid residues THR^211^, SER^215^ aromatic amino acid residue, and TYR^474^ of 1 pi~pi stacking interaction. The residue TRP^80^ between two aromatic amino acids has a separation of − 3.35A (vDw) having IUPAC name 2,5,7–trihydroxy-3-(4-hydroxyphenyl)-2,3-dihydrochromen-4-one of C_13_H_12_O_4_ (Fig. 7). After the comparison of all three models, the compound CID with 443,637 with the lowest energy is chosen for further molecular dynamics simulation studies.

The lower Glide score represents the most and highest favorable binding affinity. Hydrogen bond interactions, pi-interactions, pi staking of the best poses were visualized and interpreted using XP visualizer with descriptors (Table 7) in ascending order. It rewards the topmost ligands for hydrogen bond with lengths and angles deviating significantly from “ideal” hydrogen-bond interaction (1.65A H-A distance,180 D-H A angle) [47]. The PhobEn measures hydrophobic enclosure reward on the protein. The lipophilic EvdW is the term for the hydrophobic region that lies within receptor and Ligand proximity. For the obtained data, PiCat, ClBr, PhobEnPa, penalties, HB penal, exposed penal, zprot remained at zero, whereas other properties of descriptors were exhibited accordingly.

**Table 7:**
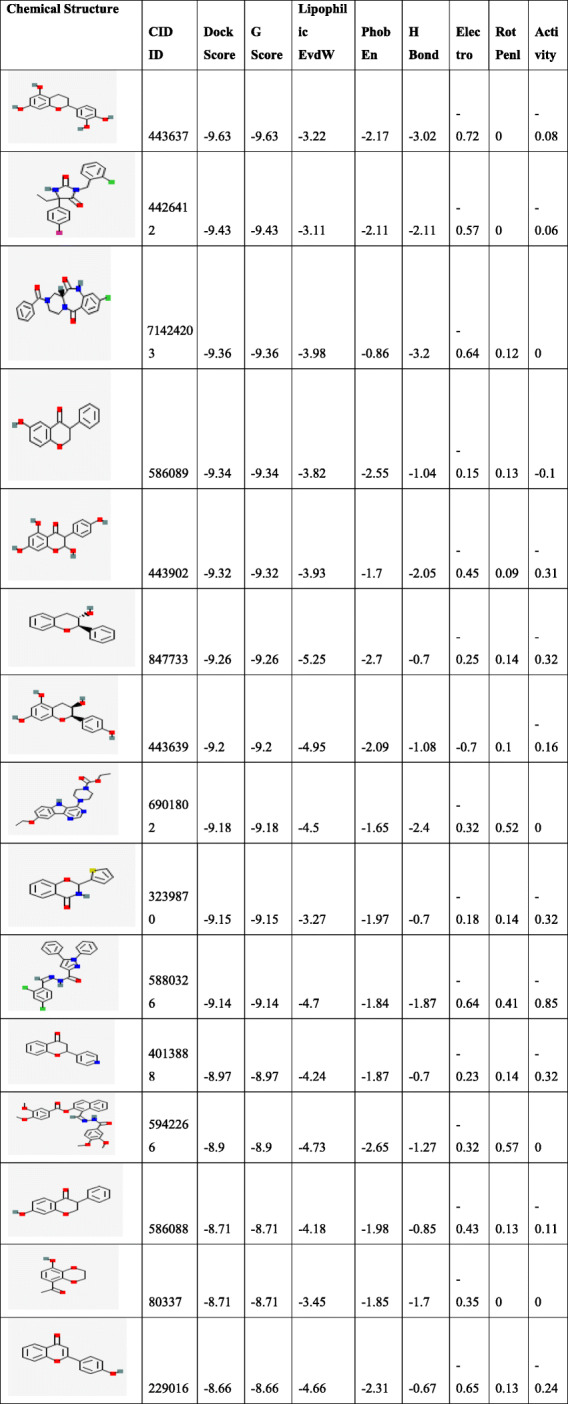
Top-ranked hit compounds of docking with protein W80R obtained using XP visualize

*G Score-total G score along with the sum of XP terms(G score = a*vdW + b*Coul + Lipo + Hbond + Metal + BuryP + RotB + Site, where vdW is van der Waals energy, Coloumb energy, Lipo is lipophilic contact, Hbond is hydrogen bonding, Metal, is metal-binding, BuryPis penalty for buried polar groups, RotBis penalty for freezing rotatable bonds, the site is polar interactions in the active site and a = 0.065 while b = 0.130 were the coefficients of vdW and Coul

Dock score: Vanderwaals + Coulombic + HBonds represent the potentiality of bonding. In simple rigid systems, the ligand is searched in a 6 dimensional rotational or translational space to fit in the binding site, which can serve as a marvel compound for drug design [64]. The lipophilic term is derived from the hydrophobic grid potential and the fraction of the total protein-ligand vdWenergy, PhobEn- can be as hydrophobic enclosure reward for the penalty for ligands with large hydrophobic contacts and low hydrogenbond scores phobic penal for the penalty for exposed hydrophobic ligand groups, Rot Penal for the rotatable bond penalty.

### Molecular dynamics simulation

**Fig. 8:**
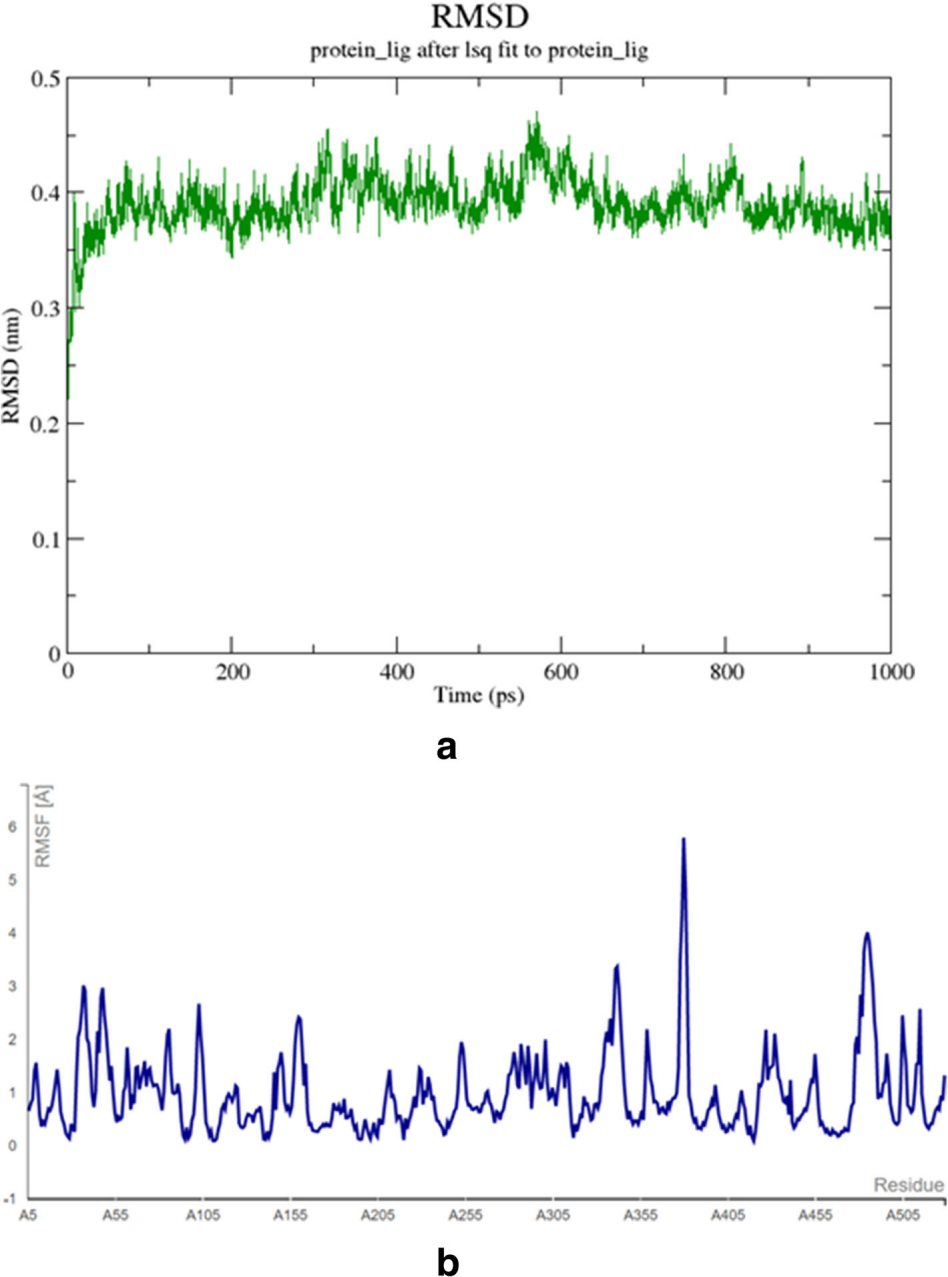
(**A**) Root mean square deviation (RMSD) of the C-alpha backbone of the W80R protein complex and ligand taxifolin (X-axis time scale in ps and Y-axis in RMSD in nm). (**B**) Root mean square fluctuation (RMSF) for C-alpha backbone atom of a W80R protein complex with ligand taxifolin (X-axis shows amino acid residue number and Y-axis shows RMSF in nm)

MD simulations were performed to W80R protein-ligand complex with the least binding energy (Fig. 8**A).** The results of MD trajectories were evaluated by the root mean square deviation (RMSD) and root mean square fluctuation (RMSF) plot which could provide significant insights into understanding structural changes in atomic details. The RMSD is a significant parameter to analyze the equilibrium in MD trajectories, which is estimated for backbone atoms of W80R protein and taxifolin ligand complex. For the W80R protein complex, the fluctuations were raised about 0.3 to 0.4 nm during the initial stage (Fig. 8**A)**. Clear and noticeable deviations were observed in the residues of RMSD values with an increase in time from 200 ps to 600 ps. The majority of residues resulted to attain a stable state at 600 psbetween 0.45 nm to 0.5 nm. At the same time, W80Rprotein-ligand complex fluctuated from 700 ps to 900 ps at 0.4 nm and remained stable between 0.4 nm to 0.45 nm until the end of the simulation [65]**.**

RMSF results were obtained by considering the average of all backbone residues of atoms to inspect the local variations of protein flexibility **(**Fig. 8**B).** The fluctuations observed above have an important role in protein complex flexibility and thus affect protein-ligand activity and stability. The high RMSF value shows more flexibility with a maximum level of fluctuation in the residue positions of 355 and 405 at 6 Å of the backbone structure, while the minimum RMSF shows very limited movements. The RMSF graph for the W80R-ligand complex was shown in Fig. 8B. The W80R-ligand complex has attained the amino acid residues at 455 and 500 also shows a fluctuation at 5 Å of RMSF. While at positions 305 and 355 at 4Å indicate a similar steep-up graph at 5 Å.The amino acid residues between 15, 55, and 105, 155, have shown medial deviation at 3 Å.

**Fig. 9:**
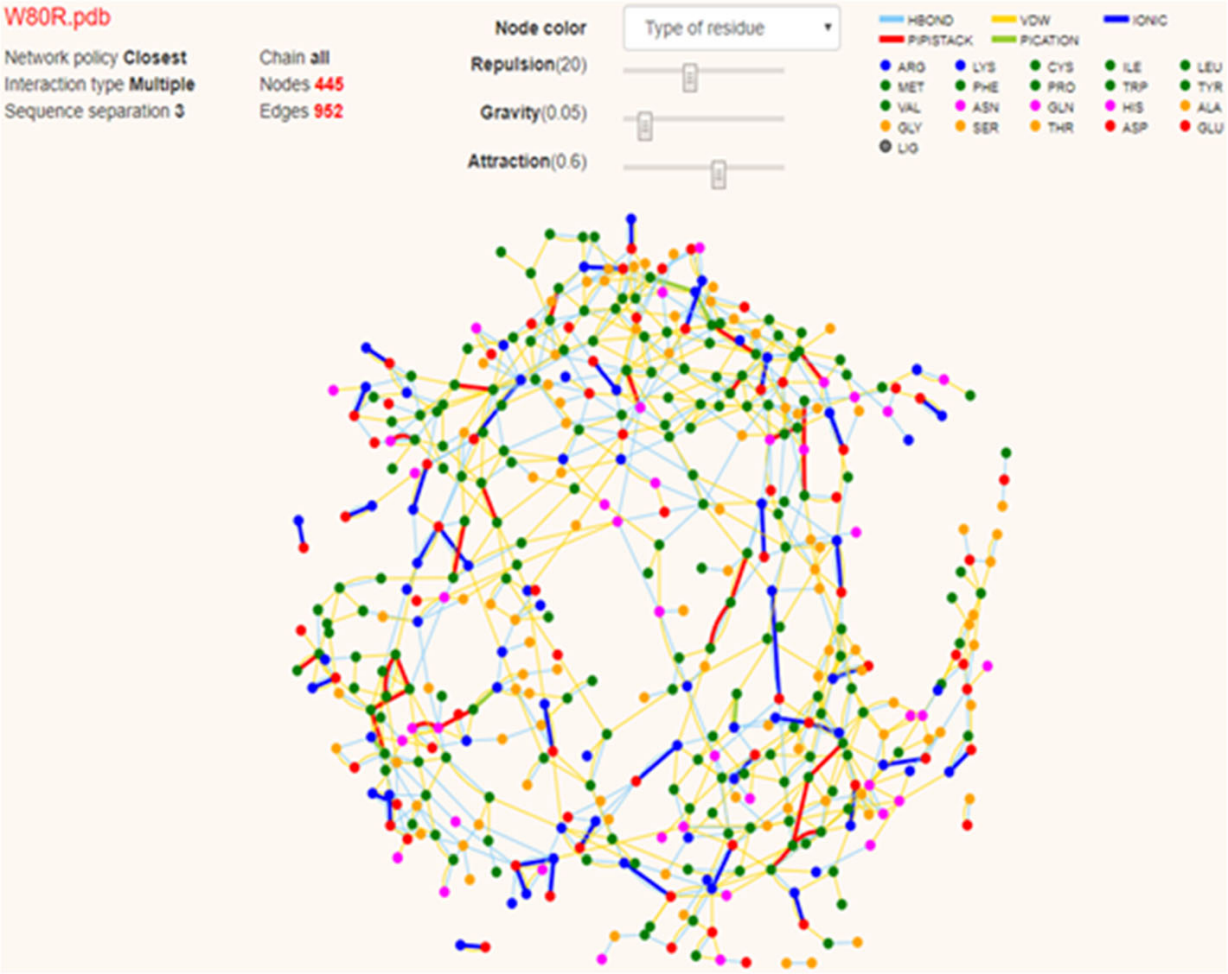
Visualisation of the residual network of a W80R protein complex

To determine the residue interaction network, RING2.0 software identifies all types of non-covalent interactions in atomic levels which have wide different energies and lengths. The output has been visualized in two different ways (i) interaction network which has been visualized using different labels and (ii) structural contacts using RING_viz-script for pymol **(**Fig. 9**).** The applications of RING 2.0 have a growth in protein folding patterns, domain-domain communication and catalytic activity, inter-intrachain interactions that combine both solvent and ligand atoms. Residue interaction network (RIN) describes the single amino acid as nodes and physicochemical properties as edges including covalent and non-covalent bonds. RIN has become common practice to explore the complexity inherent in macromolecular systems [66].

### Validation of marvel compound with AKT1-inhibitors

This study has compared the marvel compound (taxifolin) with co crystallised protein-ligand (inhibitors) complex. Among the proposed models for AKT1 protein, the best and high 3D resolution of protein 4GV1 pdb format is considered for better understanding the specificity and potency of the inhibitor when compared other authentic protein ligands further evaluation. This cocrystalised pdb molecule is subjected to auto dock programming software for evaluating binding energy, RMSD values and K_i_ (nm). The ligands were choosen and downloaded from RSCB database. The Auto dock program 4.2 [67] has been employed for this purpose which utilizes a semi- empirical free energy. The force field is based on thermodynamic model which takes into account of intermolecular energies. The difference between bound and unbound states of ligands associated with their calculations of binding energies. However, the force field employs the simple method of utilizing atomic charges. The force field assesses the binding energy of two or more water molecules by pair-wise atom interaction energy along with empirical approach to evaluate the surrounding water molecule. The results depicted that obtained marvel compound shown higher binding energy than co crystallized ligands. Therefore, it is redocked to check for the validation of model. The docking position accuracy is indicated by the shift in the position of the accuracy of the native ligand from its position in the respective complex of the co-crystallized protein and ligand. The results depicted that the high resolution protein structure 4GV1of AKT1 is used for redocking purpose with cocorysallised ligands. 4GV1 protein structure has also used check the obtained marvel compound (taxifolin). The validation studies confirmed the highest binding energy with taxifolin about − 13.94 kcal/mol and reference RMSD as 27.43 and K_i_ in (nm) as 60. 57All the other inhibitors were redocked and checked for RMSD and binding energy and results obtained as shown in the table. This provides the strong evidence that natural flavonoid has high binding energy while other synthetic inhibitors such as CQU, SMH, SM9 fallen between − 10.54 kcal/mol to − 9.33 kcal/mol range of energies. The detailed obtained values are given in the Table 8.

**Table 8:**
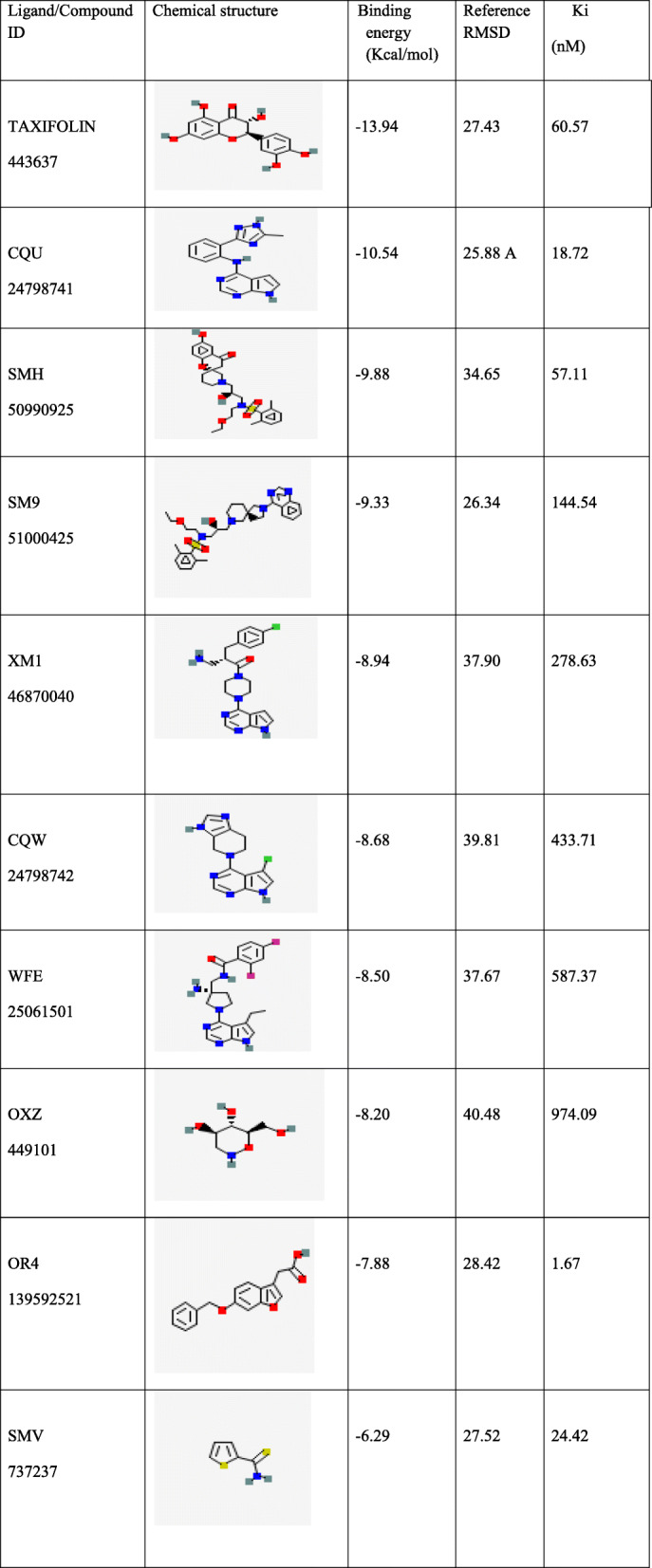
Validation of 4GV1 receptor with co crystallised inhibitors with the obtained marvel compound

Reference RMSD means the difference rms between the taken structure and the input structure. The most proper way of evaluate geometry is to measure the root means square deviation (RMSD) of a ligand from its reference position after the superimposition of the receptor molecule. It is usually considered best < 1.5–2.0 a for good accuracy

## Discussion

The enigmatic in ovarian cancer is that in nearly 75% of patients, cancer does recurse during the first two years and failto respond to available therapeutic drugs due to acquired resistance [68, 69] in addition to late diagnosis in advanced clinical stages and metastasis within the peritoneal cavity [70]. Therefore, there is an immediate need to design novel drugs to deal with the existing problem. Numerous studies since a decade have reported that Flavonoids as candidates are meant to block, retard, or reverse the progression of carcinogenesis [71]. Although various studies have been carried out using flavonoids the anticancer mechanisms have not been defined clearly. However, it was found that the flavonoids such as quercetin and silymarin induce anti-cancer mechanisms in the ovarian cancer cell [72, 73]. Consequently, the effects of apigenin, luteolin, and myricetin on ovarian cancer have to uncover the link between potential mechanisms underlying their anticancer effects. Quercetin inhibits cell proliferation of ovarian cancer cell line of SKOV-3 which correlated with findings of (Yi, 2014) caused on concentration and time-dependent manner [74] showed to inhibit UVB induced skin cancer cell proliferation and induce apoptosis in vivo models upon apigenin treatment. Taxifolin in-vitro studies have been efficient especially in anticancer, antimicrobial activities but leave a strong gap in the *invivo* studies at the root level.

The marvel compound in the current study was recognized as taxifolin which has potent to exhibit anti-cancer effects on U2OS and Saos-2 in osteosarcoma cell lines by inhibiting the proliferation and disrupting colony formation. In vivo studies exhibit intraperitoneal administration in nude mice bearing U2OS xenograft that resists tumor growth. This potency is known to arrest the G1 phase of the cell cycle in U2OS and Saos-2 cell lines. Taxifolin has known to function by inhibiting colon carcinogenesis by NF-kB mediated Wnt/b catenin signalling through upregulation of Nrf2 pathway while downregulation in genes such as TNF-α, COX-2, β-catenin, and cyclin-D1 was inhibited by NF-kB and Wnt signalling pathway [75]. It is also reported that injection of taxifolin has reduced the proliferative activity on Wistar rats with benign prostatic hyperplasia [76]**.** Taxifolin also has an excellent report on antiangiogenic effect by new blood vessels and its branches per area of chick chorioallantoic membrane assay which is inhibited by tube formation on matrigelmatrix in the human umbilical vein of endothelial cells which were evaluated against tachyzoites in vitro with IC50 of 1.39 μg/mL(*p* ≤ 0.05) along with pyrimethamine. Taxifolin has known to express an anti-proliferative effect on cancer cell types by inhibiting cell lipogenesis and inhibits the fatty acid synthesis in cancer cell lines which is able to prevent the growth of cancer cells [77].

An extensive animal (rat) study of antioxidant activity on taxifolin acid has shown the decreased lipid peroxidation in the serum and liver levels. The presence of OH groups at position 5th and 7th together with 4-OXO function in the A and C rings were meant for scavenging effect while the O-dihydroxy group in the B ring provided stability [78]**.** Consequently,In vivo studies on taxifolin induced in apoptosis of HCT116 and HT 29 cells revealed PARP1 overexpression is responsible for ovarian cancer. AKT and catenin proved that down-regulated expression by taxifolin on HCT 116 and HT 29 cells demonstrates a decline in p-AKT and catenin in a dose of 40 μM against DMSO altering in the G2 cell cycle and its regulators [79]. The expression levels of AKT, SKP-2, v-mc avian myelocytomatosis viral oncogene homolog(c-myc) and P-SER^473^, have reduced activity on AKT gene by taxifolin [80]**.** Although the above-mentioned experimental outcomes have contributed to diversified pharmacological activities with AKT1 protein, we still lack the detailed and molecular changeswrt to the W80R mutant protein of the AKT1 family. Consequently, the marginal overview of the molecular mechanism and atomic level with W80R mutation has aimed to identify hits for optimization from large data set of compounds from the PubChem database screening of flavonoids in parallel to W80R mutant protein of AKT1 targeting ovarian cancer. Table 1 for the receptor molecule W80R of 480 amino acid sequence provides detailed knowledge about the stability of protein using Protoparam tools of Expasy server. The extensive evaluation of the W80R sequence at the nucleotide level reveals its density, while other parameters such as A-T, C-G rich region, molecular weight, amino acid composition, theoretical pI, aliphatic index, instability index, and GRAVY significantly stand up for stability factor. The most favored region by RAMPAGE server was assessed to be 79.3% **(**Table 2**)** with active site binding. Furthermore, the reliability of the protein model has been assessed by 3D or homology modelling. Therefore, the Generation of 3D protein structure from sequence information, in the absence of experimentally determined structures in protein data bank through computational approaches has become the topmost priority in the scientific community based on structural biology research for several decades [81, 82]. The protein was henceforth evaluated with SAVES server (structural analysis and verification) for quality check, structural refinement through energy minimization in lowest energy state in its stable conformation, followed by ProSA **(**Fig. 1**)** and superimposition analysis with experimentally determined template structure as well as atoms and RMSD assessment to obtained a high-quality structural model for virtual screening [83, 84]**.** The predicted score for the 3D homology model of RMSD for the W80R protein was 0.18, the model was considered as the best one for further validation purposes.3D QSAR studies have been performed with structural similarity to predict the unknown/untested ligands for better potency by correlating mathematical and statistical values. QSAR models can prioritize ideas in virtual screening as well in the optimization of marvel compounds. Thus it has gained acceptance in *in-silco* drug discovery. The scatter plot QSAR tool **(**Fig. 2**)** assessed the molecular fields for the compounds which estimate the stability and establish statistical value to be 0.379 predicting the changes obtained in the training set composition with 92.7 measured higher F indicates more statistical significant regression. The dataset of 44 ligands was classified into test and training models randomly with combined mathematical and statistical approaches for the drug candidate represents phase activity of 358.477% extrapolated for 0.458 with the predicted activity of 333.692 and predicted error of − 24.7856 which was a good combination as a marvel compound. R^2^ (Coefficient of determination) value measured to be 0.822 which means the obtained model accounts for 82.2% of the variance for the observed activity, while standard R^2^ falls between 0 and 1 always (Table 6). The increase in a correlation coefficient(R^2) as an increase in the number of PLS factors, with the decrease in values of standard deviation (SD) and the increase in the number of variables, allows the user to reduce the error of the fit model. Consequently, the stability tends to increase to 3 factors, and then decrease. Here, the 3–3-model predictions were the least sensitive to the training set composition based on leave 1- out tests. But for the 4 or above factors, the R^2 larger values than stability indicates the over-fit begins at a4 factor. Of the complete training set data, the descriptor Stability indicates the model sensitivity while larger R ^2^ indicates the over-fit data set. Therefore, it is concluded that obtained data holds quite good for marvel compounds statistically. As per Lipinski’s rule of five, a drug is a good molecule if it possesses ADME (absorption, distribution, metabolism, and excretion) properties [48]**.** All the physicochemical properties and drug-likeness were listed in Tables 3, 4, and 5 consequently; it becomes easy for the marvel compound to enter the mammalian cell to interact with proteins and regulating gene expression in metabolic pathways. The top 10 hits obtained by molecular docking were further docked into the active binding sites of protein using a sitemap tool of above score 1 and grid generation followed by XP protocol (Table 7). Neverthless, a contour map is one such tool used in the present study to determine favorable regions based on field-based QSAR which depends on steric, electrostatic, hydrophobicity in solvent-accessible pockets based on least binding energy. This application plays a vital role in combination therapies of multi-drug-resistant conditions as well as in drug discovery.

The evaluated hydrophobicity gives an accurate check for the drug-ability of a compound **(**Fig. 3**).** Sitemap tool treats entire protein to locate binding sites whose size, the extent of solvent exposure is assessed based on scoring function by ranks. Active sites are ranked based on ligand propensity of binding measured by their ability to bind tightly for passively absorbed small molecules. Among the predicted combinations, active site amino acid residues of site score 1.128, drug-ability score − 1.149, volume 384.486, and size 179 **(**Fig. 4**)** were taken for further analysis. Taxifolin holds good interactions with the binding domain of W80R, highest Glide score of − 9.63 kcal/mol with O-H of GLU^234^ and H bond LEU^156^ and ASP^274^ amino acid residues and one pi-cation interaction and one hydrophobic bond with LYS^276^ (Fig. 5). The marvel molecule satisfied all the surface area calculations using QIKPROP tool of SASA, FISA, FOSA, PSAand partition coefficient of Qplogpoct, QPlogPw, QPlogPo, QPlogS, ClQPlog, QPlogHER, QPPCaco, QPPMDCK, QPlogKp, wherefore, this inhibitor of the PI3K/AKT pathway has shown diverse aptitudes for anticancer activity in both preclinical and clinical experimental values and also supported through *in-silico* analysis.

The reports by the administration of taxifolin in colorectal cancer cell lines and in the HCT 116 xenograft mouse model had shown excellent antitumor activity. The studies proved that the taxifolin hindered the mRNA expression of β-catenin thus compiling anti-proliferative activity which was arbitrated by PI3K/AKT signal by jamming Wnt/ β –catenin signaling transduction through hampering the β expression [79]**.** The elucidation of suppression by taxifolin on nuclear factor-kB, C-Fos, and mitogen-activated protein kinase also decreased osteoclast specific gene expression including Trap, Mmp-9, Cathepsin K, C-Fos, Nfatc1, and Rank; taxifolin osteoclastogenesis via regulation of many RANKL signaling pathways was also confirmed [85]. Taken together, these studies demonstrated that Wnt/catenin pathway plays a crucial role in ovarian cancer development and this idea also laid a strong platform for the development of targeted curatives.

CID- 44264122 with three hydrogen bonds of a hydroxyl group (−OH) interacting with THR^291^, ILE^290^, and THR^211^ where ILE^290^ forms oxy bond with residue LYS^268^ (Fig. 6) with Glide XP score − 9.43Kcal/mol. CID-71424203 forms the hydrogen bond interactions with residues of TYR^474^, SER^215^, THR^211^, with 1 pi-pi interactions at TRP^80^ residue, and 1 pi-cationic interaction bonding with LYS^265^ with G score − 9.36 Kcal/mol showed good hydrophobic interactions **(**Fig. 7**).** The molecular dynamics simulation was performed to obtain the lowest error and data loss. The fluctuations in relative positions of atoms in protein-ligand complex explain the structural stability (RMSD) at 0.45 nm to 0.50 nm between 600 to 800 ps **(**Fig. 8**A).** The RMSF has shown a steep up graph at 5A with a slight medial deviation and not much structural change in protein cavity was observed [86, 87] **(**Fig. 8**B).** Finally, the high resolution receptor molecule 4GV1 is used for validation purpose and estimated binding energy for the marvel compound as − 13.94 Kcal/mol after redocking. This study supported strong evidence against other synthetic inhibitors found in the database for AKT1 molecule.

Residue interaction networks (RINs) consider single amino acids as nodes and physio-chemical interactions as edges **(**Fig. 9**)** representing the protein structure as RINs have become common practice to explore the complexity inherent in macromolecular systems. Henceforth, the taxifolin has been suggested as a drug for human use in clinical trials.

Above all, assessing protein-ligand binding affinity has become the main challenge during early stages of drug discovery. Machine learning approach has been introduced in contributing to this type of prediction by two approaches. By exploring the experimental structures with binding affinity and thermodynamic data accessed using BindingDb, Binding MOAD, and PDBbind with open sources like SAnDRes and Taba, while the second method protein ligand docking simulations. Therefore this combination has outruled classical scoring functions with high predictive performance.

## Conclusions

The mutant forms of the amino acid were found to induce pathological outcomes disrupting the native conformation of a protein. The W80R mutation in the PH domain of AKT1 had been reported to cause ovarian cancer by in-vitro studies and recorded in the Cancer genome database. The clinical significance of W80R in ovarian cancer and synthetic drugs used has laid as the platform for this study. Therefore, the modelling of W80R protein has been made for the first time. To examine the detailed molecular mechanism of W80R, we conducted molecular docking along with dynamic simulation studies to understand the stability of the mutant structure, which is known to cause a damaging effect of the mutation.

Furthermore, a rise in RMSD values for stability in trajectory and conformational drifts were observed in W80R protein. The expected result supported the molecular cause in a mutant form which resulted in a gain of ovarian cancer. However, experimental evaluation or in vivo studies is recommended for further validation.

## Data Availability

Provided with the manuscript.
